# Assessment of using a gamma index analysis for patient‐specific quality assurance in Japan

**DOI:** 10.1002/acm2.13745

**Published:** 2022-08-26

**Authors:** Yusuke Anetai, Iori Sumida, Yu Kumazaki, Satoshi Kito, Masahiko Kurooka, Yoshihiro Ueda, Yuki Otani, Yuichiro Narita, Ryu Kawamorita, Kazuhiko Akita, Takahiro Kato, Mitsuhiro Nakamura

**Affiliations:** ^1^ Department of Radiology Kansai Medical University Hirakata‐shi Osaka Japan; ^2^ Department of Radiation Oncology Osaka University Graduate School of Medicine Suita‐shi Osaka Japan; ^3^ Physics and clinical support Accuray Japan K.K. Chiyoda‐ku Tokyo Japan; ^4^ Department of Radiation Oncology International Medical Center Saitama Medical University Hidaka‐shi Saitama Japan; ^5^ Department of Radiotherapy Tokyo Metropolitan Cancer and Infectious Diseases Center Komagome Hospital Bunkyo‐ku Tokyo Japan; ^6^ Department of Radiology Tokyo Medical University Hospital Shinjuku‐ku Tokyo Japan; ^7^ Department of Radiation Oncology Osaka International Cancer Institute Osaka‐shi Osaka Japan; ^8^ Department of Radiology Kaizuka City Hospital Kaizuka Osaka Japan; ^9^ Department of Medical Physics High Precision Radiation Therapy Center Aomori Shintoshi Hospital Aomori‐shi Aomori Japan; ^10^ Department of Radiation Oncology Tane General Hospital Osaka‐shi Osaka Japan; ^11^ Kansai BNCT Medical Center Osaka Medical and Pharmaceutical University Takatsuki‐shi Osaka Japan; ^12^ Department of Radiological Sciences School of Health Sciences Fukushima Medical University Fukushima‐shi Fukushima Japan; ^13^ Division of Medical Physics Department of Information Technology and Medical engineering Human Health Sciences Graduate School of Medicine Kyoto University Sakyo‐ku Kyoto Japan

**Keywords:** gamma analysis, measurement‐based patient‐specific quality assurance, multidimensional scaling, principal component analysis

## Abstract

**Purpose:**

The Task Group 218 (TG‐218) report was published by the American Association of Physicists in Medicine in 2018, recommending the appropriate use of gamma index analysis for patient‐specific quality assurance (PSQA). The paper demonstrates that PSQA for radiotherapy in Japan appropriately applies the gamma index analysis considering TG‐218.

**Materials/methods:**

This survey estimated the acceptance state of radiotherapeutic institutes or facilities in Japan for the guideline using a web‐based questionnaire. To investigate an appropriate PSQA of the facility‐specific conditions, we researched an optimal tolerance or action level for various clinical situations, including different treatment machines, clinical policies, measurement devices, staff or their skills, and patient conditions. The responded data were analyzed using principal component analysis (PCA) and multidimensional scaling (MDS). The PCA focused on factor loading values of the first contribution over 0.5, whereas the MDS focused on mapped distances among data.

**Results:**

Responses were obtained from 148 facilities that use intensity‐modulated radiation therapy (IMRT), which accounted for 42.8% of the probable IMRT use in Japan. This survey revealed the appropriate application of the following universal criteria for gamma index analysis from the guideline recommendation despite the facility‐specific variations (treatment machines/the number of IMRT cases/facility attributes/responded [representative] expertise or staff): (a) 95% pass rate, (b) 3% dose difference and 2‐mm distance‐to‐agreement, and (c) 10% threshold dose. Conditions (a)–(c) were the principal components of the data by the PCA method and were mapped in a similar distance range, which was easily clustered from other gamma index analytic factors by the MDS method. Conditions (a)–(c) were the universally essential factors for the PSQA in Japan.

**Conclusion:**

We found that the majority of facilities using IMRT in each region of Japan complied with the guideline and conducted PSQA with deliberation under the individual facility‐specific conditions.

## 1 INTRODUCTION

Intensity‐modulated radiation therapy (IMRT), including volumetric modulated arc therapy (VMAT), is a technology that realizes intricate dose distribution through complicated mechanical processes. The American Association of Physics in Medicine (AAPM) released a report on the tolerance limits and methodologies for IMRT measurement‐based verification of patient‐specific quality assurance (PSQA) known as the Task Group (TG)‐218.^1^ The American Society for Radiation Oncology ^2^, ^3^ and American College of Radiology^4^ strongly recommend IMRT PSQA as well as AAPM. PSQA is an important process for excluding unpredictable errors. If PSQA is omitted, the overconfidence bias that the machine has continuous precision of irradiation may be dangerous, leading to a tragedy as reported by the New York Times.^5^, ^6^ Furthermore, the tolerance limits and measurement methods for PSQA prior to the TG‐218 report have been completely facility‐specific. Because of the variations in treatment machines, irradiation methods (fixed or rotational), measurement devices/methods, analysis software, patient‐specific circumstances, and facility‐specific policy of treatment or QA, it is difficult to evaluate the measurement beyond the specificities. The dose difference (DD) and distance‐to‐agreement (DTA) and its combination (composite test) are basic comparisons, but the regions of low dose or steep‐gradient dose cannot be evaluated appropriately. The gamma index method proposed by Low et al.^7^ relaxes the sensitivity against failure handling. This method uses the displacement of the dose distribution between the reference $\left({\vec{r}}_{r}\right)$ and evaluated points $\left({\vec{r}}_{e}\right)$. The gamma index γ is obtained by the renormalized criteria Γ using the following equation:

(1) $$\Gamma \;\left({\vec{r}}_{e},{\vec{r}}_{r}\right)=\;\sqrt{\frac{r^{2}\left({\vec{r}}_{e},{\vec{r}}_{r}\right)}{\Delta d^{2}}+\frac{\delta^{2}\left({\vec{r}}_{e},{\vec{r}}_{r}\right)}{\Delta D^{2}}},$$

where $r({\vec{r}}_{e},{\vec{r}}_{r})$ is the distance between ${\vec{r}}_{r}$ and ${\vec{r}}_{e}$, $\delta ({\vec{r}}_{e},{\vec{r}}_{r})$ is the DD between ${\vec{r}}_{r}$ and ${\vec{r}}_{e}$, Δd represents the DTA criterion, and ΔD denotes the DD criterion. In the DD criterion, we commonly used %DD that received global normalization (divided by such as prescribed dose per fraction and maximum dose of the detection) or local normalization (divided by each dose of corresponded position). Accordingly, γ satisfies

(2) $$\gamma \;\left({\vec{r}}_{r}\right)=\min\left(\Gamma \left({\vec{r}}_{e},{\vec{r}}_{r}\right)\right)\;\forall \left({\vec{r}}_{e}\right).$$

We can address the similarity between the reference and evaluated dose distributions based on γ≤1 (pass) or γ>1 (fail). In the TG‐218 report, they clarified the criteria for measurement‐based PSQA regarding the gamma index and its passing rate as follows: (a) the universal tolerance limit is a 95% passing rate, and (b) the universal action limit is a 90% passing rate, and both (a) and (b) are under the condition of 3% DD (ΔD=3%) with 2‐mm DTA (Δd=2mm) in the 10% threshold dose distribution. The measurement method is also recommended as the true composite (TC) method that simulates the treatment delivery almost precisely, including radiation attenuation by the couch. The stationary device placed on the treatment couch detects full radiation beams using the actual treatment beam geometry, including monitor units, gantry, collimator, couch angles, and leaf positions of the multi‐leaf collimator. This report also indicates several factors pertaining to gamma index analysis: (a) The gamma failure points distributed in the clinically irrelevant region may be neglected. In contrast, the gamma failure points that are clinically relevant, such as the planning target volume (PTV) or organs at risk (OARs), should be reverified or thoroughly discussed. (b) A device that is not suitable for detecting TC should consider the method of perpendicular field‐by‐field (PFF). (c) The perpendicular composite (PC) method should not be used for PSQA because of the tendency of masking the delivery errors. (d) Absolute dose detection is recommended rather than relative dose detection. (e) Global normalization is deemed more clinically relevant than local normalization, whereas local normalization is useful and stringent for troubleshooting or commissioning. (f) The dose threshold is useful for excluding clinically irrelevant low‐dose regions.

Gamma index analysis is useful and convenient and is recommended by TG‐218. Although a comprehensive understanding of its use is required, the aforementioned facility‐specific variations, including the treatment machines, measurement devices or methods, calculation algorithms for dose distribution and their resolutions, criteria for gamma index analysis (DD, DTA, and threshold of sample points), and analysis software (interpolation and searching algorithm for 2D/3D space), remain facility‐dependent, which is a primary issue. Under these circumstances, the evaluation of the measurement and the assurance of clinical treatment are entrusted to individual facilities in Japan. We investigated the usage and appropriateness of gamma index analysis in PSQA in Japan following the published TG‐218 guidelines with multivariate analyses for the questionnaire survey, expecting to reveal underlying relationships and the essence of the complex data^8^ for an actual validity of the guideline.

## 2 MATERIALS AND METHODS

A survey comprising 46 questions (shown in the Supporting Information section) was designed to evaluate facility‐specific information in PSQA and the use of gamma index analysis. The web‐based questionnaire was developed using Google Forms (Google LLC, Mountain View, CA, USA). The survey was mainly informed by the Japanese Society of Medical Physics, a community of medical physicists in Japan, using an email with a link to the online survey. The responses were obtained from radiation oncologists, medical physicists, or radiation therapists from radiotherapeutic facilities. The survey was conducted from 12 February 2021 to 31 March 2021. Answers were obtained from 148 facilities (42.8%) conducting IMRT out of 346 facilities with possible IMRT usage in Japan after the formal notification.^9^ To assess the influence, we particularly focused on the following specific representative features of TG‐218: the gamma index analyses for global normalized 3%/2‐mm (DD/DTA) criteria, 10% threshold, and 95% pass rate. The number of TC measurement corresponding to the representative occupation (as answered in the questionnaire) and the facility attributes under specific conditions of the gamma index analysis was also investigated. The data are presented in Tables 1, 2, 3 for analysis. We estimated the appropriate dataset using the following equation^10^:

(3) $$\begin{array}{c} n_{sample}=N\left(\frac{z^{2}p\left(1-p\right)}{\varepsilon^{2}}\right)/\left(\frac{z^{2}p\left(1-p\right)}{\varepsilon^{2}}+N-1\right), \end{array}$$

where $n_{sample}$ is the ideal sample size for a *z*‐score corresponding to a certain confidence interval (CI) of a Gaussian distribution, ε is the error value, *p* is the sample proportion, and *N* is the population size. The error term ε is therefore specifically expanded as follows:

(4) $$\begin{array}{ccc} \varepsilon & = & \sqrt{\frac{\left(N-n\right)}{n\left(N-1\right)}z^{2}p\left(1-p\right)}\\ & = & \sqrt{\frac{198}{148\times 345}\times 1.96^{2}\times \left(\frac{148}{346}\right)\times \left(\frac{198}{346}\right)}≅0.060, \end{array}$$

where the substitutions are *z* = 1.96 (95% CI), *N* = 346, *n* = 148, and p= 148/346. For this moderate amount of data, we can presume the valuable features of the majority of the population through multivariate analyses.

**TABLE 1:** Participant facility attributes, staff, and relevant QA individuals for the survey

Facility attributes			Participants
University hospitals			43
Cancer center hospitals			10
National hospital organizations and public hospitals			38
Other organization hospitals			57
Total number of valid responses (facilities): 148			

Occupations	Measurement	Analysis and evaluation	Clinical approval
Medical doctor	0	3	21
Medical physicist	99	107	115
Radiation therapist	131	95	64
Radiotherapy quality manager	69	58	47
Other	1	0	1
Total number of valid responses (facilities): 148			

Number of relevant people	Measurement	Analysis and evaluation	Clinical approval
1	13	32	52
2	42	46	55
3	19	15	19
4–5	33	28	14
6–9	34	23	8
10–	7	4	0
Total number of valid responses (facilities): 148			

**TABLE 2:** Main gamma index analysis methods employed with facility‐specific factors

			DD/DTA			PR		Dose TH		
Manufacturer	Machine	*n*	3%/2 mm	3%/3 mm	2%/2 mm	95%	90%	10%	30%	50%
Varian (87 facilities)	TrueBeam STx/Edge	23	15	3	4	14	7	16	2	0
	TrueBeam	47	30	9	4	28	14	35	1	2
	Halcyon	3	2	1	0	0	2	2	0	0
	VitalBeam	1	0	1	0	0	1	1	0	0
	Clinac iX series	51	29	12	9	33	18	36	6	4
	Clinical EX series	7	3	4	0	4	3	4	0	2
Elekta (35 facilities)	Unity	0	0	0	0	0	0	0	0	0
	VersaHD	15	9	2	3	10	3	13	1	0
	Infinity	5	3	2	0	3	2	2	2	0
	Synergy	23	12	4	6	14	8	11	2	1
Siemens (5 facilities)	ARTISTE	0	0	0	0	0	0	0	0	0
	ONCOR	5	1	2	2	3	1	3	2	0
	PRIMUS	0	0	0	0	0	0	0	0	0
Accuray (31 facilities)	Radixact	12	6	3	3	8	4	5	2	0
	Tomo HD A	3	1	2	0	1	2	1	0	1
	Tomo HD	9	5	3	0	5	4	4	0	1
	Tomo H	3	0	3	0	1	2	1	1	0
	CK S7	0	0	0	0	0	0	0	0	0
	CK M6	7	5	2	0	4	3	3	1	2
	CK VSI	2	1	1	0	1	1	1	0	1
	CK G4	1	0	1	0	1	0	0	0	0
	CK G3	1	0	1	0	1	0	1	0	0
Other (15 facilities)	Novalis	10	5	2	2	4	4	7	1	0
	Vero4DRT	4	2	2	0	2	2	2	0	0
	MRIdian	2	1	1	0	1	1	2	0	0
Total number of valid responses (facilities): 148										

			DD/DTA			PR		Dose TH		
	Number of cases	*n*	3%/2 mm	3%/3 mm	2%/2 mm	95%	90%	10%	30%	50%
Maximum annual number of IMRT treated cases (2015–2020)	1–49	21	11	6	4	15	5	13	3	3
	50–99	33	21	3	6	19	13	23	4	2
	100–199	49	28	10	9	36	11	32	4	4
	200–299	25	13	7	3	14	9	15	1	1
	300–399	11	5	3	1	4	6	6	1	0
	400–499	2	0	2	0	0	1	1	0	1
	500–749	3	2	1	0	0	3	2	1	0
	750–999	1	0	1	0	1	0	0	0	0
	1000–1499	2	2	0	0	2	0	2	0	0
Total number of valid responses (facilities): 147, one is invalid answer										

			DD/DTA			PR		Dose TH		
	Factor	*n*	3%/2 mm	3%/3 mm	2%/2 mm	95%	90%	10%	30%	50%
Institute/facility	University hospitals	43	25	9	5	26	16	29	4	2
	Cancer center hospitals	10	7	0	2	6	3	6	0	0
	National hospital organization/public hospitals	38	20	10	8	24	13	20	5	4
	Other organization hospitals	57	30	15	8	35	17	39	6	5
Expertise/staff	Medical physicist	72	42	12	13	43	25	47	6	5
	Radiation technologist (therapist)	69	39	18	9	44	22	45	8	4
	Radiotherapy quality manager	7	1	4	1	4	2	2	1	2
Total number of valid responses (facilities): 148										

Abbreviations: CK, CyberKnife; DD, dose difference; DTA, distance‐to‐agreement; IMRT, intensity‐modulated radiation therapy; PR, pass rate; TH, threshold.

**TABLE 3:** Number of choices in patient‐specific quality assurance (PSQA) measurements and gamma index analysis for specific scenarios

				Hospital attributes				Respondents		
Gamma analysis criteria (DD/DTA) (*n*)	Gamma index analysis criteria (threshold and pass rate)	*n*	TC method use (*n*)	UH	CC	NPH	OH	MP	RT	QM
3%/2 mm (82)	TH 10%, 95% pass rate	39	31	13	5	9	12	19	20	0
	TH not 10%, 95% pass rate	16	15	4	0	5	7	8	8	0
	TH 10%, not 95% pass rate	17	15	4	0	3	10	9	7	1
	TH not 10%, not 95% pass rate	10	8	4	2	3	1	7	3	0
3%/3 mm (34)	TH 10%, 95% pass rate	12	11	2	0	3	7	5	7	0
	TH not 10%, 95% pass rate	8	6	1	0	4	3	2	3	3
	TH 10%, not 95% pass rate	6	4	3	0	1	2	2	4	0
	TH not 10%, not 95% pass rate	8	7	3	0	2	3	3	3	1
2%/2 mm (23)	TH 10%, 95% pass rate	11	9	3	1	2	5	7	3	1
	TH not 10%, 95% pass rate	1	1	0	0	1	0	1	0	0
	TH 10%, not 95% pass rate	5	4	2	0	2	1	3	2	0
	TH not 10%, not 95% pass rate	6	6	0	1	3	2	2	4	0
Other (9)	Included any conditions	9	7	4	1	0	4	4	4	1
Total number of valid responses (facilities): 148										

Abbreviations: CC, cancer center hospital; DD, dose difference; DTA, distance‐to‐agreement; MP, medical physicist; NPH, national hospital organizations and public hospital; OH, other organization hospital; QM, radiotherapy quality manager; RT, radiation therapist; TC, true composite; TH, threshold; UH, university hospital.

We deemed the summarized values in Table 2 as the numerical features for this survey that could become a numerical factuality for the facility‐ and machine‐dependent PSQA in Japan. Dependent or independent variables for multivariate analysis are controversial,^11^ and both cases should be evaluated. In this study, we introduce two representative methods for analysis. Principal component analysis (PCA) was applied to factor decomposition for linear correlated relations. Multidimensional scaling (MDS),^12^, ^13^, ^14^ a manifold learning method of machine learning, was also applied to data feature analysis for nonlinear relations regardless of correlation. The MDS method effectively maps the similarity of each data node based on the Euclidean or geodesic distance. Accordingly, this method reveals the nonlinear and underlying relationships among the data nodes.^15^, ^16^, ^17^, ^18^, ^19^, ^20^ The factors for analysis were all composed of the number of facilities; therefore, we did not apply standardization (subtracting mean and dividing by standard deviation; *z*‐score) but normalization (dividing by 148 that is the total facility number using IMRT in this survey) for preprocessing. We used scikit‐learn (version 1.0.2) for the PCA and MDS calculations,^21^ a widely used Python 3.8 module library for machine learning.

## 3 RESULTS

### 3.1 Information of the participant facilities

The responses were obtained from 148 out of 346 radiotherapy facilities with a clinical use of IMRT in Japan. The breakdown of the participants regarding facility attributes or responding staff and the number of people for PSQA are shown in Table 1. Main gamma index analytic criteria employed with those facility‐specific factors (treatment machines/the maximum annual number of treated cases using IMRT/facility attributes/expertise or staff) are demonstrated in Table 2. Table 3 shows the number of choices in the specific gamma index analytic criteria scenarios for the measurement method (TC), facility attributes, and responding staff. Tables 4, 5, 6 indicate the detailed responses in the questionnaire regarding dose calculation, dosimetry, and evaluation of PSQA. The Supporting Information section also provides the details of data of the questionnaire (Tables S1–S44).

**TABLE 4:** Dose calculation information of participant facilities

Part 1					
Number of items	Questionnaire items		Number of facilities		
(Suppl. Table number)	Main items	Auxiliary items	Non‐SRT	SRT	Valid responses
#01 (S8)	Most used dose calculation algorithm for a clinical plan	Pencil‐beam	3	2	148
		Superposition	84	70	
		Monte Carlo	60	65	
		No specific items	1	11	
	Most used dose calculation algorithm for a QA plan	Pencil‐beam	3	4	148
		Superposition	85	74	
		Monte Carlo	59	59	
		No specific items	1	11	
#02 (S9)	Most used dose specification (dose reporting) for a clinical plan	Dose‐to‐water	59	49	148
		Dose‐to‐medium	73	76	
		No specific items	16	23	
	Most used dose specification (dose reporting) for a QA plan	Dose‐to‐water	73	64	148
		Dose‐to‐medium	58	59	
		No specific items	17	25	
#03 (S10)	Most used CT slice for dose calculation for a clinical plan	<1.0 mm	0	2	148
		≥1.0, <1.5 mm	8	70	
		≥1.5, <2.0 mm	11	8	
		≥2.0, <2.5 mm	85	45	
		≥2.5, <3.0 mm	35	13	
		>3.0 mm	8	0	
		No specific resolution or not for clinical use	1	10	
	Most used CT slice for dose calculation for a QA plan	<1.0 mm	2	5	148
		≥1.0, <1.5 mm	23	57	
		≥1.5, <2.0 mm	12	9	
		≥2.0, <2.5 mm	74	49	
		≥2.5, <3.0 mm	29	17	
		>3.0 mm	7	1	
		No specific resolution or not for clinical use	1	10	
#04 (S11)	Most used dose calculation grids for a clinical plan	<1.0 mm	2	4	148
		≥1.0, <1.5 mm	7	45	
		≥1.5, <2.0 mm	9	10	
		≥2.0, <2.5 mm	86	55	
		≥2.5, <3.0 mm	32	18	
		>3.0 mm	11	5	
		No specific resolution or not for clinical use	1	11	
	Most used dose calculation grids for a QA plan	<1.0 mm	2	5	148
		≥1.0, <1.5 mm	11	43	
		≥1.5, <2.0 mm	13	12	
		≥2.0, <2.5 mm	86	56	
		≥2.5, <3.0 mm	26	18	
		>3.0 mm	9	3	
		No specific resolution or not for clinical use	1	11	

Part 2				
Number of items	Questionnaire items		Number of facilities		
(Suppl. Table number)	Main items	Auxiliary items	Non‐SRT	SRT	Valid responses
#05 (S12)	Most used gantry spacing resolution for a clinical plan	<1.0°	2	4	148
		≥1.0°, <1.5°	21	21	
		≥1.5°, <2.0°	81	74	
		≥2.0°, <2.5°	2	2	
		≥2.5°, <3.0°	8	6	
		>3.0	11	10	
		No specific resolution or not for clinical use	23	31	
	Most used gantry spacing resolution for a QA plan	<1.0°	2	4	148
		≥1.0°, <1.5°	21	21	
		≥1.5°, <2.0°	81	73	
		≥2.0°, <2.5°	2	2	
		≥2.5°, <3.0°	8	6	
		>3.0°	11	10	
		No specific resolution or not for clinical use	23	32	
#06 (S13)	Clinically tolerable number of coplanar static fields in IMRT planning	5 fields or 6 fields	22	10	148
		7 fields or 8 fields	37	36	
		9 fields	18	12	
		10 fields	8	7	
		11 fields or more	13	14	
		Not for clinical use	50	69	
	Clinically tolerable number of non‐coplanar static fields in IMRT planning	5 fields or 6 fields	17	5	148
		7 fields or 8 fields	24	39	
		9 fields	13	12	
		10 fields	7	12	
		11 fields or more	13	17	
		Not for clinical use	74	63	
#07 (S14)	Clinically tolerable number of coplanar arc fields in VMAT planning	1 arc	5	5	148
		2 arcs	34	23	
		3 arcs	39	30	
		4 arcs	25	28	
		5 arcs or more	19	22	
		Not for clinical use	25	40	
	Clinically tolerable number of non‐coplanar arc fields in VMAT planning	1 arc	2	3	148
		2 arcs	16	9	
		3 arcs	24	15	
		4 arcs	24	27	
		5 arcs or more	20	30	
		Not for clinical use	62	64	

Abbreviations: CT, computed tomography; IMRT, intensity modulated radiation therapy; QA, quality assurance; SRT, stereotactic radiation therapy; VMAT, volumetric modulated arc therapy. As related information, see Tables S8–S11 (part 1) and Tables S12–S14 (part 2).

**TABLE 5:** Dosimetry information of participant facilities

	Questionnaire items			
Number of items (Suppl. table number)	Main items	Auxiliary items	Number of facilities	Valid responses
#01 (S17)	Measurement devices for a point absorbed dose in the region of interest	Ionization chamber dosimeter (0.5–0.6 cm^3^)^a^	42	148
		Ionization chamber dosimeter (0.1–0.2 cm^3^)^a^	52	
		Ionization chamber dosimeter (0.01–0.1 cm^3^)^a^	36	
		Semiconductor dosimeter	1	
		Multiple dimensional dosimeter	16	
		No measurement	1	
#02 (S18)	Measurement devices for a dose distribution	2D array detector	15	148
		Cylindrical array detector	107	
		3D dose detector	1	
		Film	23	
		No measurement	2	
#03 (S19)	Measurement devices for an intensity (fluence map)	EPID	39	148
		Gantry‐mounted detector	4	
		Other detector	1	
		No measurement	104	
#04 (S23)	The most clinical interest point for the measurement of patient‐specific QA	Center of phantom	75	148
		Center of PTV	55	
		Point of interest in PTV	100	
		Center of OAR	12	
		Point of interest in OAR	63	
		Other	7	
#05 (S24)	Calculation dose for the measurement of patient‐specific QA	Volume average dose	112	148
		Point dose	34	
		Not for clinical use	2	
#06 (S25)	Most used slab‐type phantoms for the measurement of patient‐specific QA	Tough Water/Tough Lung (Kyoto Kagaku, Co. Ltd.)	60	132 (missing value: 16)
		Solid Water/Solid Water HE (Gammex, Inc.)	29	
		Virtual Water/Blue Water (Standard Imaging Inc.)	10	
		Other	7	
		Not for clinical use	26	
	Most used multipurpose phantoms for the measurement of patient‐specific QA	I'mRT Phantom (IBA Dosimetry GmbH)	46	148
		Delta4 (ScandiDos, AB.)	31	
		RT 3000 New‐Water (R‐TECH, Inc.)	13	
		ArcCHECK (Sun Nuclear Corporation)	11	
		IMRT Phantom (Standard Imaging, Inc.)	9	
		Virtual Water (Cheese Phantom) (Accuray Inc.)	6	
		Other	22	
		Not for clinical use	10	
#07 (S28)	Additional auxiliary equipment/device/fixture for dose calculation	Treatment couch	136	148
		Fixtures influencing an accuracy of radiation (Vac‐Lok etc.)	30	
		Aids for supporting patient posture (Toweling mat etc.)	9	
#08 (S29)	Setting‐up method for the measurement of patient‐specific QA	Use of localizing laser	142	148
		Use of imaging‐guided technique	40	
		No particular actions	2	
		Other actions	5	
#09 (S30)	Mainly evaluated purpose of the patient‐specific QA	Dose distribution	140	148
		Radiation intensity (fluence map)	7	
		No evaluation for both of them	1	

^a^ The ionization chamber dosimeters are categorized as regular‐ (0.5–0.6 cm^3^), mini‐ (0.1–0.2 cm^3^), and micro‐types (0.01–0.1 cm^3^).

Abbreviations: EPID, electronic portal imaging device; IMRT, intensity modulated radiation therapy; OAR, organ at risk; PTV, planning target volume; QA, quality assurance. As related information, see Tables S17–S19, S23–S25, and S28–S30.

**TABLE 6:** Evaluation information of participant facilities regarding patient‐specific quality assurance (PSQA)

	Questionnaire items		Number of facilities		
			Main purpose for dosimetry		
Number of items (Suppl. table number)	Main items	Auxiliary items	Dose distribution	Intensity (fluence map)	Valid responses
#01 (S31)	Evaluation criteria for DD	3%	95	4	147 (invalid answer: 1)
		2%	20	1	
		1%	0	0	
		Not for clinical use	24	2	
		Other	1	0	
#02 (S32)	Evaluation criteria for DTA	3 mm	31	0	147 (invalid answer: 1)
		2 mm	77	4	
		1 mm	2	1	
		Not for clinical use	28	2	
		Other	2	0	
#03 (S33)	Evaluation criteria for gamma analysis	3%/2 mm	77	4	147 (invalid answer: 1)
		3%/3 mm	33	1	
		2%/2 mm	23	0	
		2%/1 mm	1	1	
		Not for clinical use	6	0	
		Other	0	1	
#04 (S34)	Evaluation threshold for gamma analysis	10%	89	5	147 (invalid answer: 1)
		20%	17	0	
		30%	15	0	
		40%	3	0	
		50%	10	0	
		Not for clinical use	5	0	
		Other	1	2	
#05 (S35)	Reference (or rationale) dose for the gamma analysis to evaluate the dosimetric result in the evaluated region or in the whole dose distribution	Fractionated prescribed dose	45	2	145 (invalid answer: 1) (missing value: 2)
		Dose at corresponded points	45	2	
		Dosimetric maximum dose	27	2	
		Percentage of dosimetric maximum dose	7	1	
		Average of dosimetric dose	4	0	
		Maximum dose in a region of interest	1	0	
		Average dose of a region of interest	3	0	
		Not for clinical use	4	0	
		Other	2	0	
#06 (S36)	Evaluation pass rate for gamma analysis	85%	0	0	147 (invalid answer: 1)
		90%	48	1	
		95%	87	3	
		98%	0	1	
		Not for clinical use	1	1	
		Other	2	1	

Abbreviations: DD, dose difference; DTA, dose‐to‐agreement. As related information, see Tables S31–S36.

Out of 148 facilities, 53 (35.8%) cancer centers and university hospitals used IMRT in this survey. The annual largest number of treated patients of over 100 cases via IMRT was 63.3% of the 147 facilities (one responded invalid answer). Medical physicists and radiation therapists equally contributed to PSQA in measurement or analysis/evaluation, whereas medical doctors raised their number in the clinical quality of the treatment plan and its approval. The most frequent number of relevant people who performed measurement, analysis, or clinical approval was two.

Treatment machines in the participant facilities were from Varian (Varian Medical Systems Inc., Palo Alto, CA): 87 (58.8%); Elekta (Elekta Oncology Systems Inc., Crawley, UK): 35 (23.6%); Siemens (Siemens Healthineers AG, Erlangen, Germany): 5 (3.4%); Accuray (Accuray Inc., Sunnyvale, CA, USA): 31 (20.9%); and other manufacturers: 15 (10.1%), as shown in Table 2. The number of C‐arm versus O‐ring linacs of the machines in the participant facilities was 199 versus 33. There were also the variations of treatment planning systems (TPS) for radiotherapy in the participant facilities (multiple responses were allowed); specifically, Eclipse (Varian Medical Systems): 98; Monaco (Elekta Oncology Systems): 44; RayStation (RaySearch Laboratories AB, Stockholm, Sweden): 37 (25.0%); Precision (Accuray): 18; MultiPlan (Accuray): 6; iPlan (Brainlab AG, Munich, Germany): 39; Pinnacle (Phillips healthcare Inc, Andover, MA): 35; and XiO (Elekta Oncology Systems): 14, respectively. Most facilities used the same TPS manufacturer for the treatment machine and owned a TPS from a different manufacturer. Therefore, we focused on the number of treatment machines in this study.

### 3.2 Dose calculation and dosimetry information of the participant facilities

The number of facilities corresponding to the most used calculation algorithm, specification or reporting, CT slice interval, dose calculation grids, gantry spacing resolution, and clinically tolerable number of fields and arcs are shown in Table 4. The most preferred calculation algorithm, CT slice interval, calculation grids, and gantry spacing resolution were the superposition algorithm, both more than 2.0 mm but less than 2.5 mm, and more than 1.5° but less than 2.0°, regardless of the stereotactic radiation therapy (SRT)/non‐SRT or clinical/QA plan (Table 4, #01**–#**05). Many participating facilities selected coplanar VMAT for IMRT; in contrast, the coplanar/noncoplanar static‐field technique had been abandoned for clinical use by many facilities (Table 4, #06, #07). The measurement devices in the region of interest for absorbed dose, dose distribution, and radiation intensity are shown in Table 5. A mini‐type ionization chamber (∼0.1 cm^3^) detector is mostly preferred for point absorbed dose in 52 (35.1%) facilities, followed by the regular‐type ionization chamber detectors in 42 (28.4%) and micro‐type ionization chamber dosimetry in 36 (24.3%) facilities (Table 5, **#**01). Cylindrical array detectors are popular for dose distribution in 107 (72.3%) facilities, whereas film dosimetry and 2D array detectors are considered in 23 (15.5%) and 15 (10.1%) facilities, respectively (Table 5, **#**02). The measurement of radiation intensity (fluence map) was mostly performed by electronic portal imaging device in 39 (26.3%) facilities; whereas the gantry‐mounted type was noted in only four (2.7%) facilities. No measurement or assessment was noted in the radiation intensity of 104 (70.3%) facilities (Table 5, **#**03). In contrast, most of the facilities measured and assessed the dose distribution and point absorbed dose (Table 5, **#**02, #09). No measurement or assessment was noted in only one facility for point absorbed dose detection and two facilities for dose distribution. The most commonly used measurement method for PSQA is TC, followed by PC, and PFF; these methods were used in 124 (83.8%), 15 (10.1%), and 8 (5.4%) facilities (Table 3), respectively, except for one facility that answered invalidly. The most clinically interesting point for the phantom measurement considered to be appropriate was a point of interest in PTV (100), the center of the phantom (75), a point of interest in OAR (63), the center of the PTV (55), the center of the OAR (12), and others (7) (Table 5, **#**04). In the comparison method for dose measurement and calculation, 112 (75.7%) facilities adopted volume‐averaged dose in the region of interest, whereas a total of 34 (23.0%) facilities adopted trusted point doses (Table 5, **#**05). Phantoms and material‐correction methods for patient‐specific QA measurement in the types for slab or multipurpose measurements are shown in Table 5, **#**06. A multipurpose‐type phantom was preferred because of measurement efficiency. In the QA dose calculation process in TPS, a treatment couch was considered in 136 (92.0%) facilities. In contrast, fixtures or aids for supporting the patient posture possibly affected the accuracy, for example, Vac‐Lok cushions (CIVCO Medical Solutions, Orange City, IA) or toweling mat were considered in 30 and 9 facilities, respectively (Table 5, **#**07). To set up the measurement system for PSQA, almost all facilities used localizing laser. Moreover, 40 facilities considered image‐guided process for the accuracy (Table 5, **#**08).

### 3.3 Gamma index analysis information of the participant facilities

**FIGURE 1:**
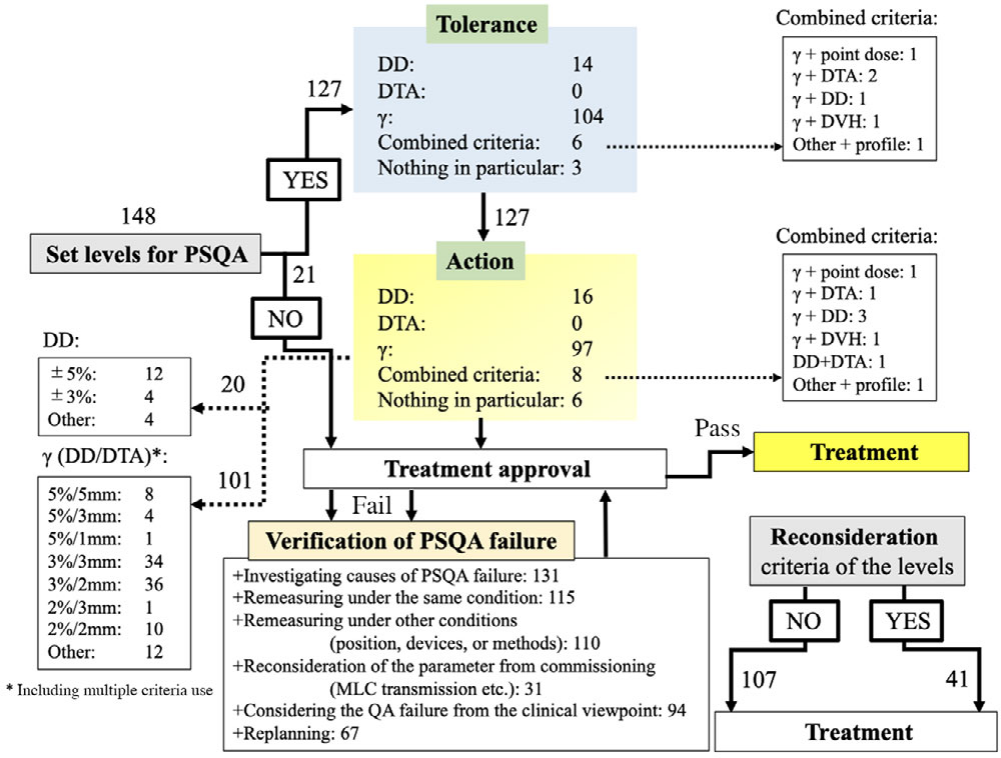
Breakdown of the facility tolerance and action levels against the patient‐specific quality assurance (PSQA). The flow shows the 127 out of 148 facilities that set the tolerance and action levels. Regarding the action level, 20 facilities evaluated dose difference (DD) and 101 facilities conducted gamma index analysis based on the shown criteria. Even if the treatment approval is not achieved due to the PSQA failure, the verification process is shown in the figure. Reconsideration of the criteria for the tolerance or action levels was considered by 41 facilities.

Evaluations for dose verification in the participant facilities are shown in Table 6. The verification of the dose distribution was considered to be important in the QA process for almost all (140, 94.6%) facilities. In contrast, a few (7, 4.7%) facilities considered radiation intensity or fluence maps as sufficient verification for the patient‐specific QA. Many facilities in Japan considered the accuracy for dose, including scattered radiation in the phantom, whereas a few facilities prioritized time‐efficiency from their robust machine quality. Of 147 facilities, 99 facilities selected 3% DD (Table 6, **#**01), and 81 facilities chose the 2 mm DTA criteria (Table 6, **#**02). In the gamma index analysis, compared with the selection of 3%/3 mm (81) in DD/DTA, 3%/2 mm (34) was selected more than 2.3 times (Table 6, **#**03). Many facilities evaluated optional DD/DTA analysis in addition to gamma index analysis. The 10% dose threshold and 95% pass rate criteria were mainly preferred (in 94 and 91 facilities, respectively), compared with the other criteria, which are shown in Table 6 **(#**03**–#**06). Table 2 shows the primary use of the criteria of gamma index analysis and the corresponding treatment machines, number of patient IMRTs, attributes of the hospital, and QA staff who responded to this survey. Most participants used Varian treatment machines and have had over 100 clinical cases for IMRT in a year. The gamma index analysis under specific scenarios based on the factors of TC measurement, facility attributes, or staff is shown in Table 3. A rationale dose for evaluation (reference dose for DD or a determination of appropriateness for the dosimetry) was selected based on the type of logic, as shown in Table 6, **#**05. The two main rationales behind the selected specific doses were the prescribed dose per fraction or dose at the corresponding points in the evaluated region of the entire dose distribution. AAPM TG‐218 and the guideline on the physical and technical aspects for IMRT from Japanese Society for Radiation Oncology were referenced in the majority of the facilities (64.8% and 83.8%), as shown in Table 7, where the former and the latter recommends 3%/2‐mm and 3%/3‐mm DD/DTA criteria for gamma index analysis, respectively. Most of the facilities (85.8%) introduced tolerance/action level. The gamma index analysis criterion was popular in 104 and 97 facilities pertaining to the tolerance and action levels in 127 facilities that set the levels for patient‐specific QA. Among them, 15 or 20 facilities considered DD analysis for the evaluation of tolerance or action levels. The breakdown of the criteria and the flow of PSQA are shown in Figure 1. To cope with the gamma index analysis failure, 131 (88.5%) of the 148 facilities investigated the causes of failure and 94 (63.5%) considered QA failure from a clinical point of view (Figure 1). For violations of the action level, TG‐218 also recommends remeasurement with a different detector or different geometries. In this survey, most facilities enforced remeasurement under other conditions about positions, devices, or methods (74.3%). Only 37 (25.0%) of the 148 facilities evaluated the predicted patient‐inner dose (Table 8, **#**01**–#**03), regardless of whether the gamma failures could affect the clinically relevant dose. However, 16 of these facilities performed dose–volume histogram analysis for the patient‐inner dose, which is still an unfamiliar method in many facilities for PSQA (Table 8, **#**03).

**TABLE 7:** Referenced documents for patient‐specific quality assurance (QA)

References	Year	Organization	*n*
Task Group 218	2018	AAPM	96 (64.8%)
Task Group 119	2009	AAPM	69 (46.6%)
Task Group 100	2016	AAPM	22 (14.9%)
Medical Physics Practice Guideline 5.a.	2015	AAPM	20 (13.5%)
Guideline for QA system of external radiation therapy	2016	JASTRO	79 (53.4%)
Guideline for physical and technical matters for IMRT	2011	JASTRO	124 (83.8%)
Guideline for IMRT accuracy using multi‐leaf collimator	2004	JASTRO	59 (39.9%)
Guideline for stereotactic body radiotherapy	2006	JASTRO	78 (52.7%)
Other references (study meetings, academic conferences, data from other facilities)	–	–	79 (53.4%)
Total number of valid responses (facilities): 148			

Abbreviations: AAPM, American Association of Physicists in Medicine; IMRT, intensity modulated radiation therapy; JASTRO, Japanese Society for Radiation Oncology.

**TABLE 8:** Predicted patient‐inner dose evaluation of participant facilities

	Questionnaire items			
Number of items (Suppl. Table number)	Main items	Auxiliary items	Number of facilities	Valid responses
#01 (S21)	Consider or not consider predicted patient‐inner dose distribution	Yes No	29 119	148
#02 (S22)	Software for predicted patient‐inner dose evaluation	PerFRACTION	5	148
		3DVH	21	
		Mobius3D	1	
		Compass	1	
		Dosimetry Check	1	
		Other	0	
#03 (S37)	Evaluation criteria for predicted patient‐inner dose	DD	2	148
		DTA	0	
		*γ*	18	
		DVH	16	
		Nothing in particular	111	
		Other	1	

Abbreviations: DD, dose difference; DTA, distance‐to‐agreement; DVH, dose‐volume histogram. As related information, see Tables S21–S22 and S37.

### 3.4 PCA and MDS analyses

**FIGURE 2:**
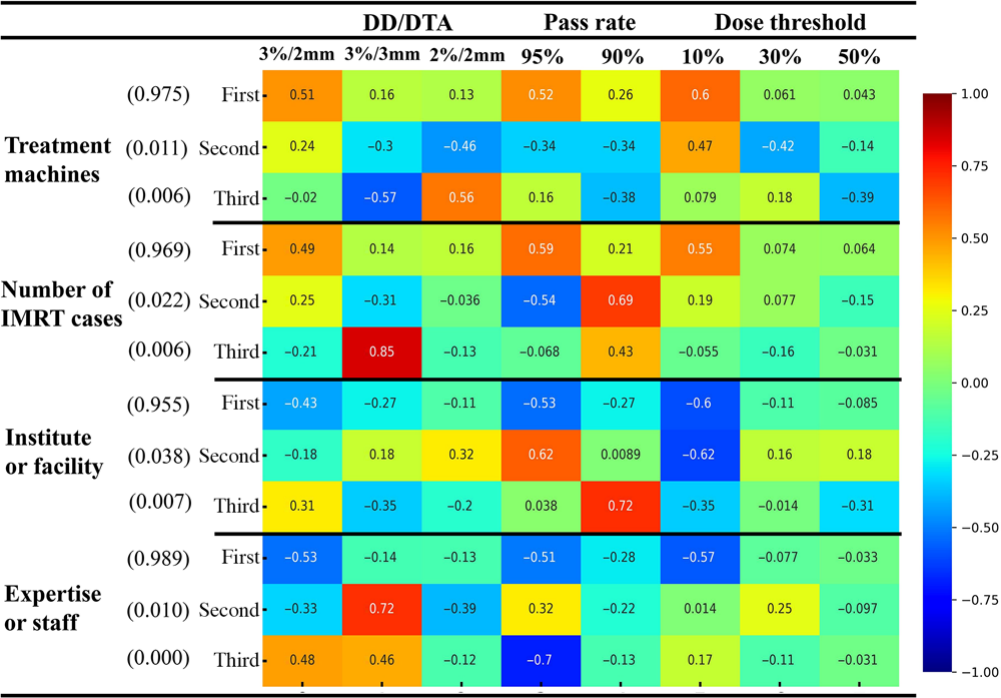
Heatmap for principal component analysis (PCA) analysis against the factors denoted by the normalized value of each column of Table 2 divided by 148 (total number of facilities using intensity‐modulated radiation therapy [IMRT]). Ordered principal component (first, second, and third) with its contribution ratio (value) are also shown. The color scale represents factor loading and correlation for the principal axes.

**FIGURE 3:**
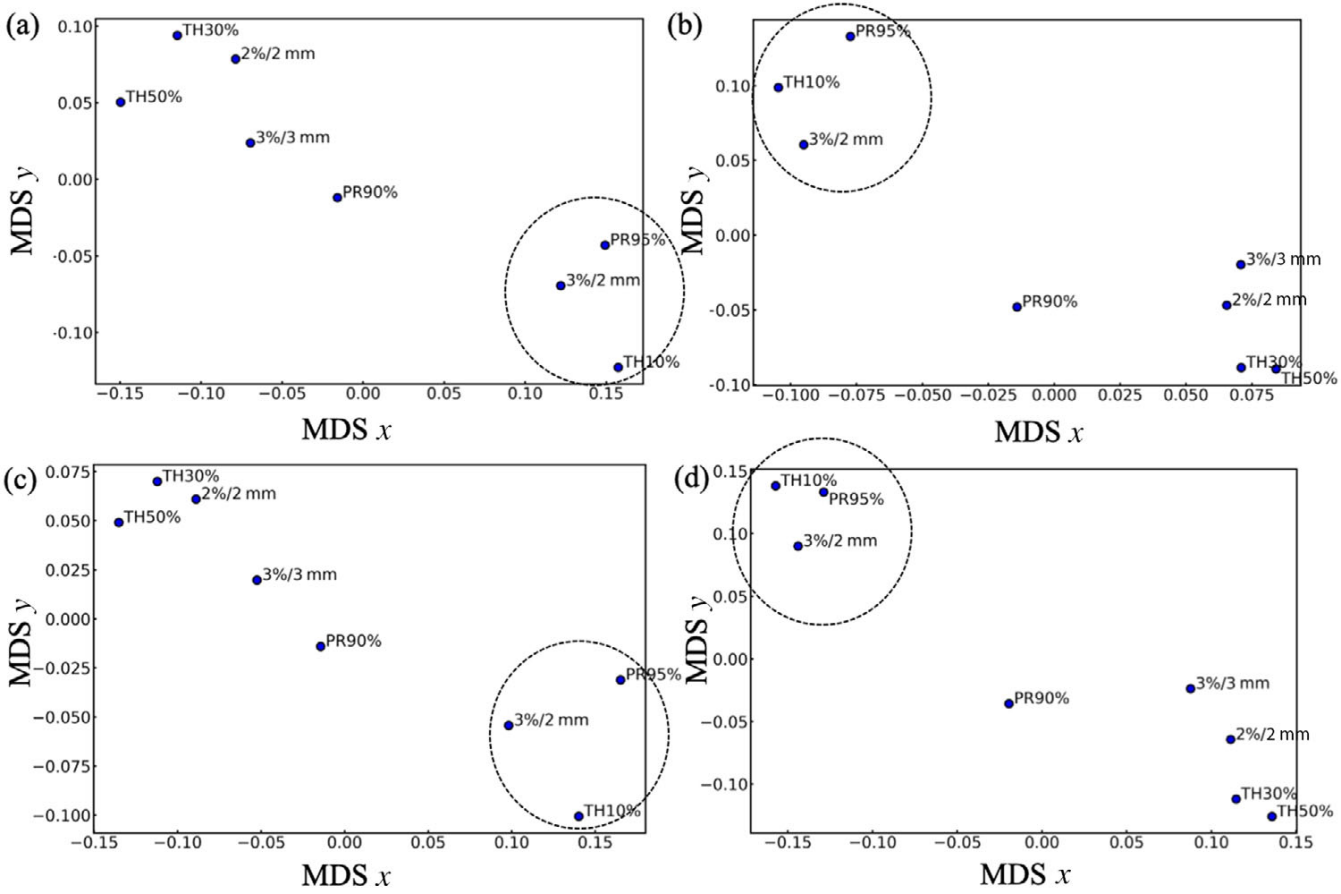
Multidimensional scaling (MDS) analysis for the factors shown in Table 2. Distance for each node is calculated using the values of Table 2 normalized by 148 (total number of facilities using intensity‐modulated radiation therapy [IMRT]). MDS *x*‐ and *y*‐axis represent similarity for each node. (a) Treatment machines, (b) maximum number of annual cases for IMRT (2015–2020), (c) institute or facility attributes, and (d) responded expertise or staff

The comprehensive factors of TG‐218 were evaluated as principal components and similarity against the conditions of gamma index analysis as the factors. These have been mainly adopted in the facilities: DD/DTA (3%/2 mm, 3%/3 mm, or 2%/2 mm), 95% or 90% pass rates for the analysis, and dose threshold (10%, 30%, or 50%). As shown in Figure 2, the first principal axis accounted for more than 90% of the weight. The color scale represents the factor loadings, indicating correlations for the principal component axes. The criteria of 3%/2 mm, 95% pass rates, and 10% dose threshold were the principal variables from their factor loading value for the data regarding any facility‐specific conditions shown in Figure 2. In contrast, as shown in Figure 3, the MDS *x*‐ and *y*‐axis represent the degree of similarity. The representative conditions of TG‐218, 3%/2 mm, 95% pass rate, and a 10% dose threshold were well clustered, despite the facility‐specific factors presented in Table 2. Therefore, the conditions of TG‐218 were widely approved and applied irrespective of the facility‐specific backgrounds, such as the treatment machines, the maximum number of IMRT cases, institute or facility attributes, staff representative (questionnaire respondent), or expertise. Although Table 3 shows that only 31 (20.9%) facilities appeared to follow the TG‐218 recommendation (95% pass rates at 3%/2 mm, 10% threshold analysis condition in TC measurement) thoroughly, they employed TG‐218 optimized conditions considering the abovementioned facility‐specific characteristics.

## 4 DISCUSSION

This survey aims to evaluate the adoption of the gamma index analysis of PSQA in Japan after the publication of the TG‐218 guidelines. PSQA is an important process for patient safety. However, the facility‐specific aspects of the machines, clinical policy, staff, measurement system, and evaluation complicate the patient‐specific assurance condition and acceptability. Apart from these circumstances, it is challenging to evaluate the clinical accuracy of PSQA via gamma index analysis because gamma index analysis permeates the ambiguity of the criteria of DD/DTA, referred dose for DD, evaluation dose threshold, pass rate, local or global comparison, 2D or 3D search, dose calculation (grid size, algorithm, and specification/reporting ), spatial resolution for the detection, and interpolation of evaluation points. TG‐218 reviewed various reports on PSQA and proposed an optimal gamma verification metric: pass rate exceeding 95% as the universal tolerance level under the condition of measurement via the TC method, 3%/2 mm, and 10% dose threshold, and pass rate exceeding 90% as the universal action level under the condition of measurement via the TC method, 3%/2 mm, and 10% dose threshold.

Nakamura et al. surveyed the actual situation of Japanese radiotherapy using IMRT in 2012.^22^ The report revealed inadequate manpower for PSQA, such as deployment of medical physicists or access to full‐time worked QA expertise. The tolerance and action levels for PSQA were already known at the time of the last survey, and gamma index analysis was conducted in 90% of the participating facilities; however, the concept was on the verge of becoming important and a 90% pass rate with 3%/3‐mm gamma index analysis influenced by TG‐119^23^ was common at that time. The situation in Japan has improved over time: Staff expertise for PSQA has improved, and the number of medical physicists has grown 1.8 times from 729 to 1337 (2012–2021). Moreover, owing to the publication of TG‐218, facilities using IMRT have set a clearer and improved standard for PSQA measurement and analysis. This survey indicates that 3%/2 mm, 10% dose threshold, and 95% pass rate are the standard in Japan. The tolerance/action level has also been set in more than 85% of the facilities in this survey. Measurement tools have also evolved, and both the efficacy and effectiveness of PSQA have improved. Furthermore, a more accurate dose calculation algorithm in TPS, rapid calculation time of optimization and dose distribution, and IMRT commissioning methodology have been established. These improvements facilitate the application of IMRT in a wider variety of complex clinical cases. Facilities with more than 200 patient cases annually treated with IMRT were rarely found at the time of the last survey. In contrast, one third of the facilities achieved this number in this survey. Moreover, most of the facilities appropriately understood the facility‐specific circumstances, and thus, they efficiently and effectively performed PSQA.

The limitation of this survey is not the sufficient but moderate sample size for statistical evaluation (sample proportion of 42.8% from 346 facilities). Our sample size is equivalent to allowing 6.0% errors at 95% confidence levels as indicated by Equation (4). We used a multivariate analytical approach on the data. This technique is generally applied to marketing research and can reveal data features from complex data. We employed PCA and MDS methods for the overall evaluation of the typical factors of TG‐218. Figures 2 and 3 reflect the 3%/2 mm (DD/DTA), 95% pass rate, and 10% dose threshold as the principal conditions that indicate strong similarity despite the variation of Tables 4, 5, 6. These results revealed that the TG‐218 concept was widely accepted in most radiotherapeutic facilities in Japan with its correct understanding. These facts also strengthen the validity of the PSQA concept recommended by TG‐218 despite the variation of the facility‐specific situation, which could also be the validity of the application to another region outside Japan whose number of annual IMRT cases over 100 (63.3% ± 6.0%) is similar or greater. Although TG‐218 clarified the orientation for PSQA and recommended the universal criteria of the gamma index, the attitude for the measurement failure is still required. In the perspective of the process‐base concept of TG‐218,^1^ the gamma index analysis is required to match clinical irradiation accuracy with each facility's circumstances. In that sense, it can be said that the necessary and sufficient PSQAs based on the TG‐218 concept was conducted by the majority of the facilities considering time‐consuming work, machine performance, and staff skills.

In addition, another perspective on whether gamma failure is clinically relevant is required.

Clinically relevant/irrelevant gamma failures require further evaluation in the difference between the phantom and patient body, because the error does not always yield a radiobiological difference.^24^ When satisfied or failed gamma index analysis in homogeneous phantom, the required modulated dose distribution is not always achieved and reproduced in vivo especially in the irradiation under the strong modulated intensity required by complicated mechanical process.^25^ Recently, a calculation‐based QA guideline emerged.^26^ The PSQA approach in Japan will develop further to standardize physical quantities.^27^ However, the concept of patient safety and its assurance for treatment must remain unchanged. The evaluation of the PSQA has to be universal and uniform for all facilities; the gamma index evaluation is certainly convenient and widely used, but the actual evaluation allows a variety of parameters. Although each facility has its own clinical policy, this study is valuable in that it indicates whether the TG‐218 guideline has been accepted and appropriately applied for the actual PSQA in Japan.

## 5 CONCLUSION

PSQA is an important process for patient safety, and evaluating an actual measurement based on the scheduled treatment plan should be appropriately performed under complex conditions. The guideline for PSQA published by AAPM in 2018 provides a standard evaluation using gamma index analysis for the measurement dose. However, these criteria require appropriate understanding and usage. We investigated the actual situation in Japan using a questionnaire and obtained the responses of 148 facilities using IMRT (6.0% errors at 95% confidence levels). The survey revealed the wide acceptance of the TG‐218 recommendations (3%/2 mm, 10% dose threshold, 95% pass rates, and tolerance/action level) and improvements (machines, staff, the number and variety of clinical cases, and accuracy) relative to the last survey of 2012 in Japan. Based on the analyses of the previous factors using PCA and MDS, most radiotherapeutic institutes or facilities in Japan carefully devised the conditions of PSQA to match their equipment, clinical policy, and treatment situation. TG‐218 is well referenced and well accepted by the radiotherapeutic facilities, and its PSQA concept for actual IMRT use has matured in Japan.

## AUTHOR CONTRIBUTIONS

Mitsuhiro Nakamura conceptualized the project and managed the study. Yusuke Anetai collected and analyzed the data. Iori Sumida advised data analysis and machine learning method. Yu Kumazaki, Satoshi Kito, Masahiko Kurooka provided clinical expertise and advised the questionnaire items. Yusuke Anetai wrote the first draft of the manuscript. Mitsuhiro Nakamura revised the manuscript. Yoshihiro Ueda, Yuki Otani, Ryu Kawamorita, Kazuhiko Akita, and Takahiro Kato checked the validity of the analysis and supervised the manuscript. All coauthors approved the final manuscript.

## CONFLICT OF INTEREST

The authors declare that there is no conflict of interest that could be perceived as prejudicing the impartiality of the research reported.

## Supporting information

Supporting Information
