# Supplementary material for: Assessment of using a gamma index analysis for patient‐specific quality assurance in Japan
Source: J Appl Clin Med Phys. 2022 Aug 26;23(10):e13745. doi: 10.1002/acm2.13745 (PMC9588274; doi:10.1002/acm2.13745)
Supplement: Supplementary file 1 — Supporting Information [file ACM2-23-e13745-s001.docx]

**Assessment of using a gamma index analysis for patient-specific quality assurance in Japan (Supplement Data: Questionnaire results)**

**Table S1. Number of actual treatments with all radiotherapy or IMRT (Q1–Q2)**

**
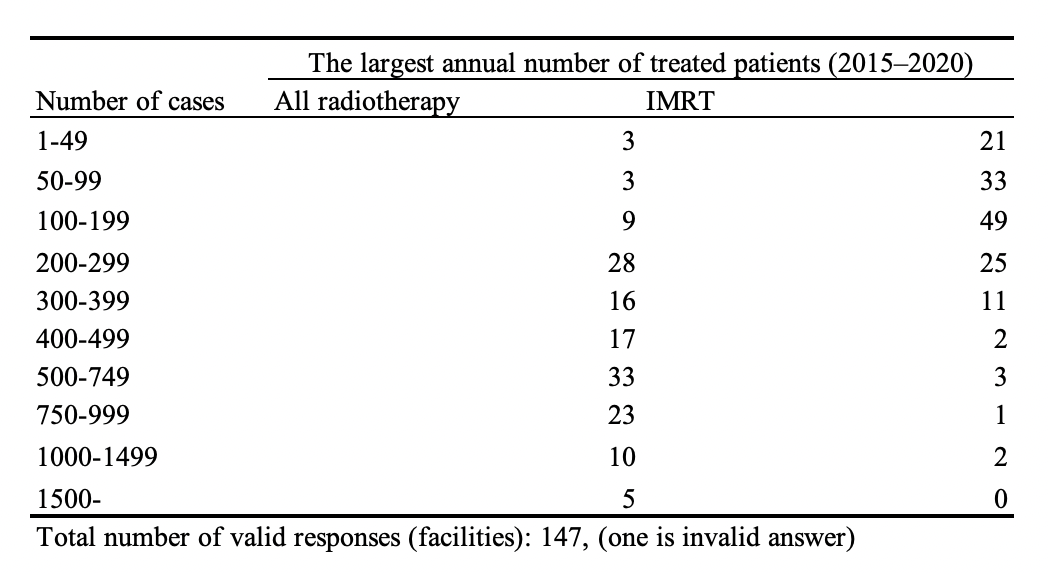
**

**Table S2. Relevant occupation and the number of people for patient-specific QA (Q3–Q4)**

**
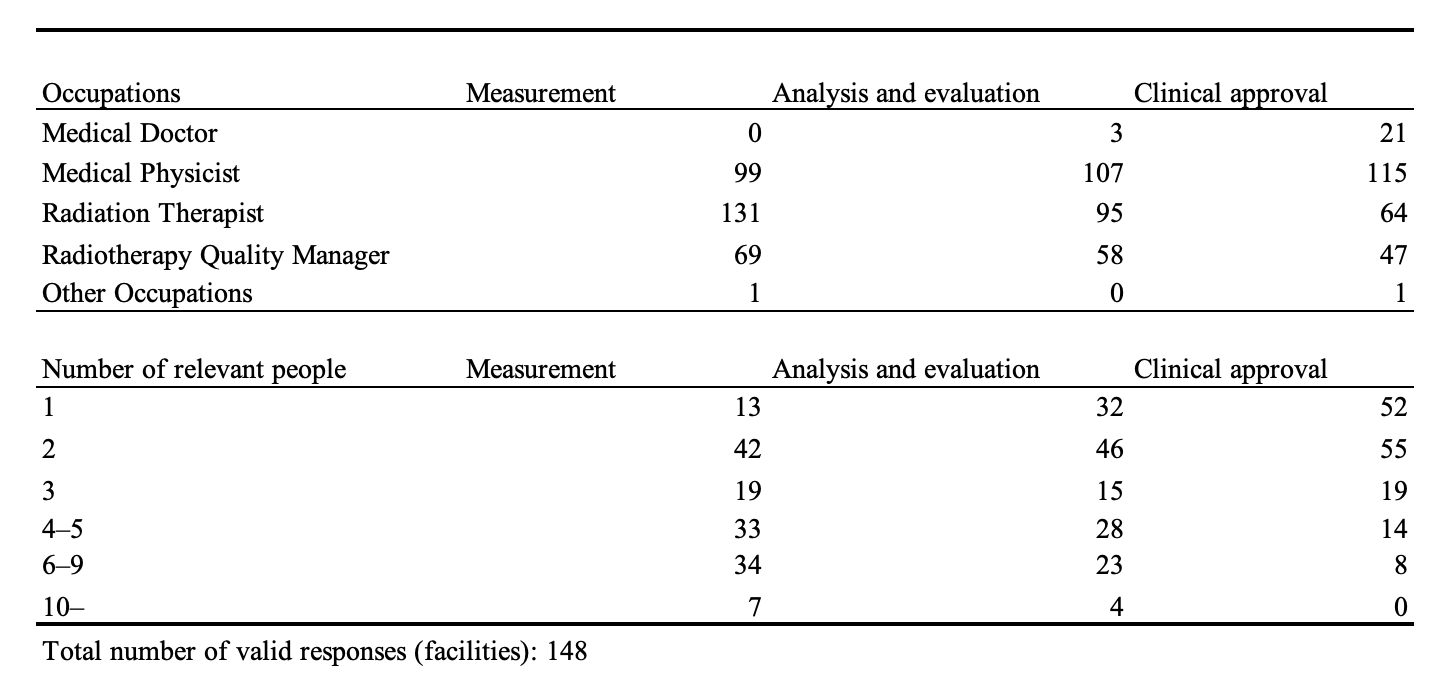
**

**Table S3. Coping methods for the deviation from the facility criteria (Q5)**

**
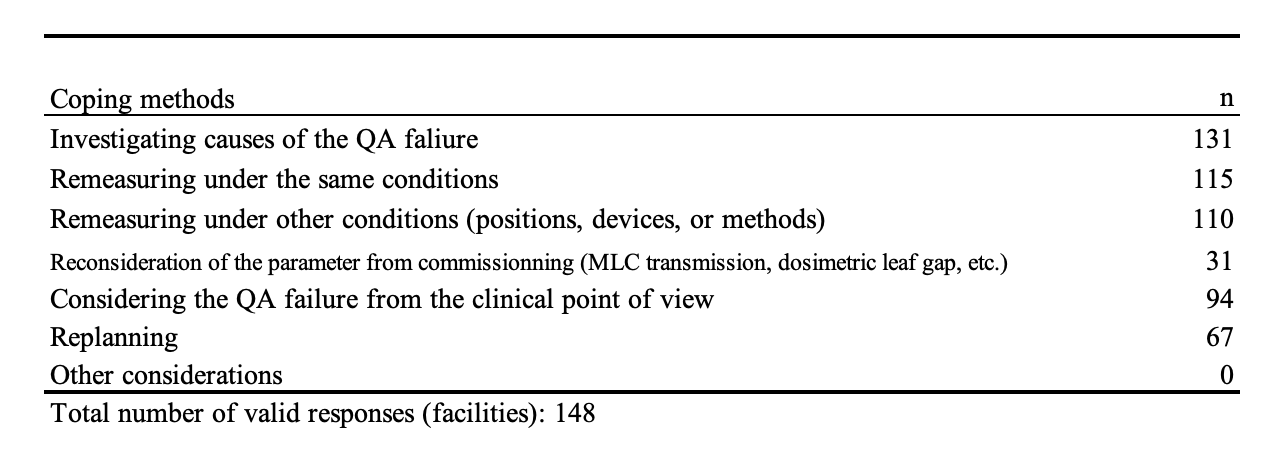
**

**Table S4. Rationales for clinical approval and the relative occupations (Q6)**

**
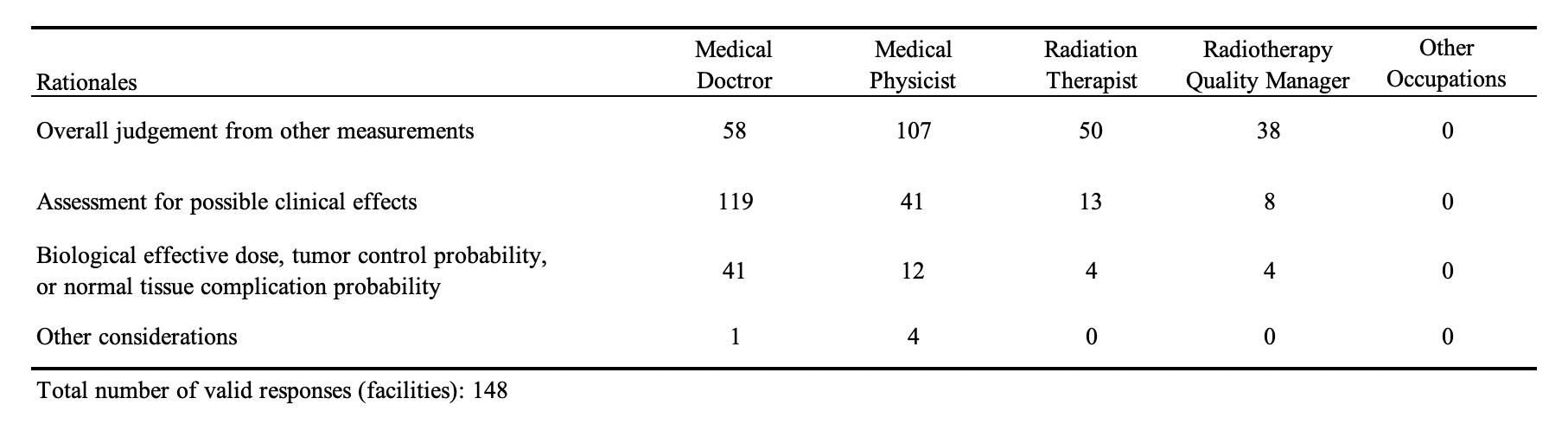
**

**Table S5. Treatment machines for radiotherapy (including multiple units to be owned) (Q7)**

**
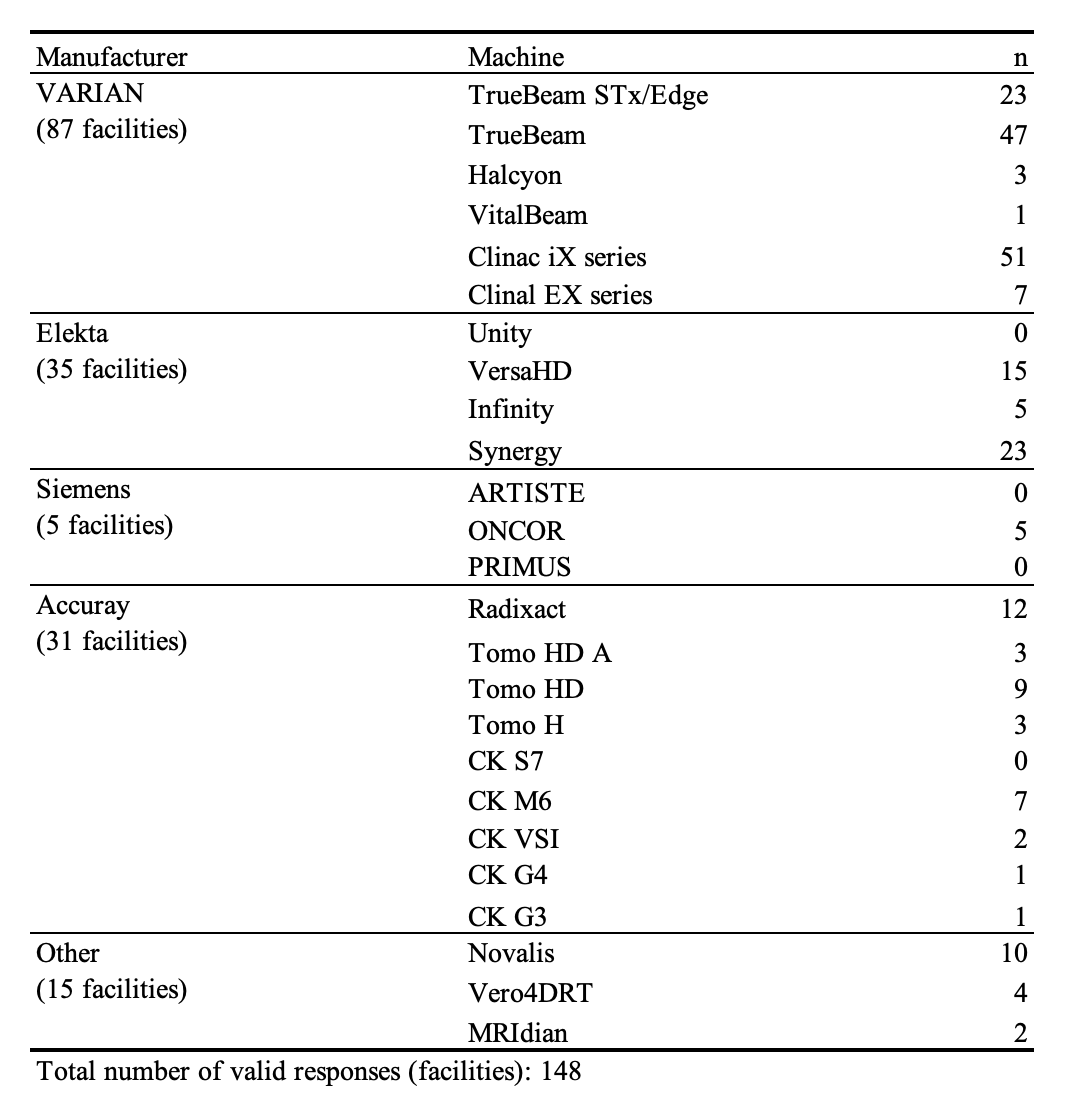
**

**Table S6. Treatment planning system (including multiple units to be owned) (Q8)**

**
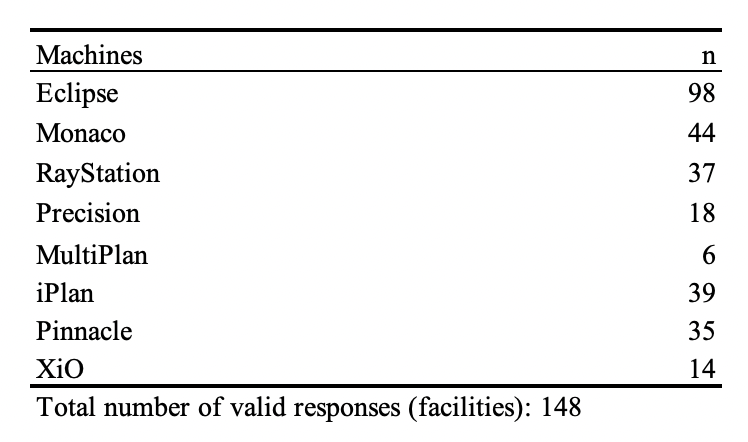
**

**Table S7. Image-guiding methods for radiotherapy (Q9)**

**
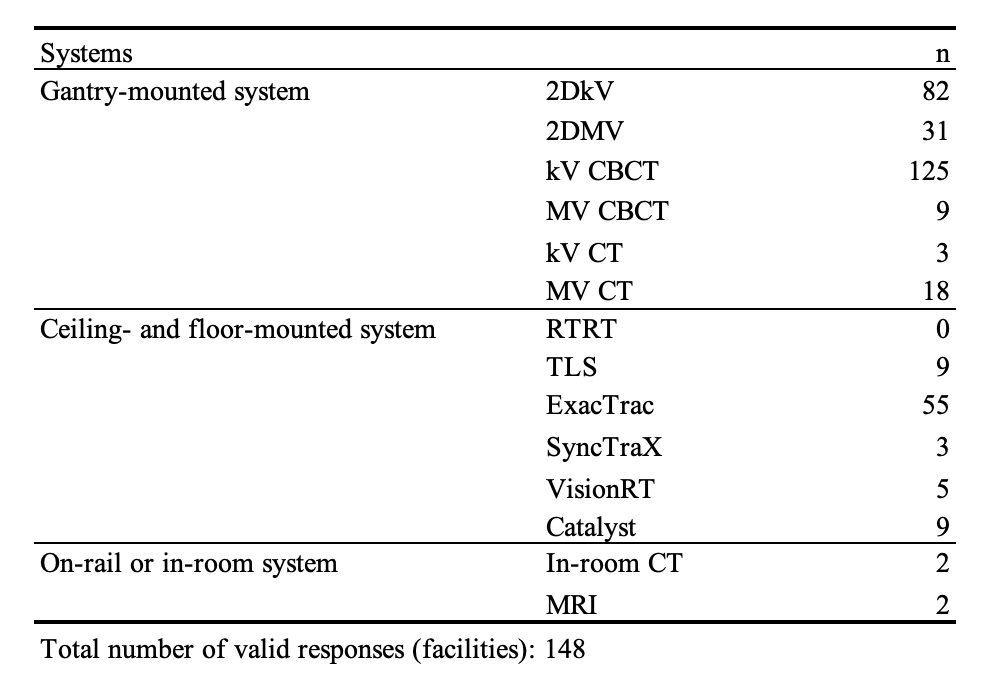
**

**Table S8. Most used algorithm for dose calculation in IMRT (Q10)**

**
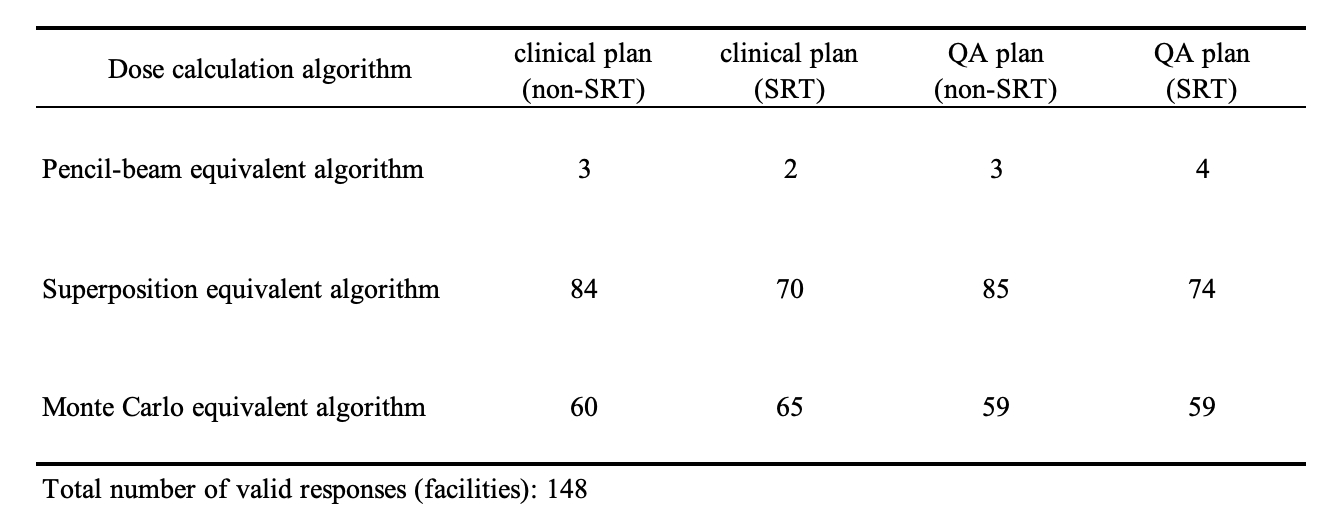
**

**Table S9. Most used dose specification or reporting method (Q11)**

**
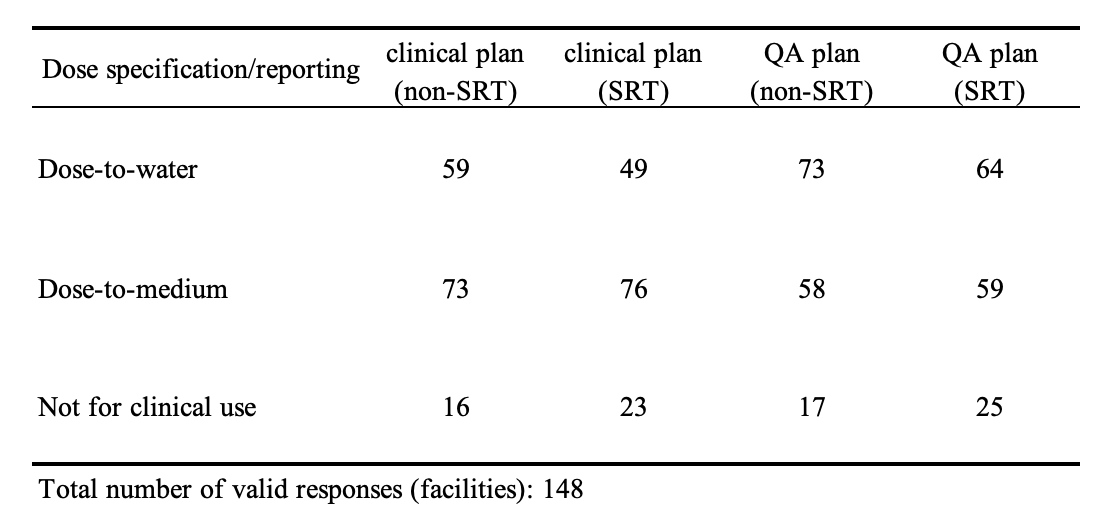
**

**Table S10. Most used CT slice interval for dose calculation (Q12)**

**
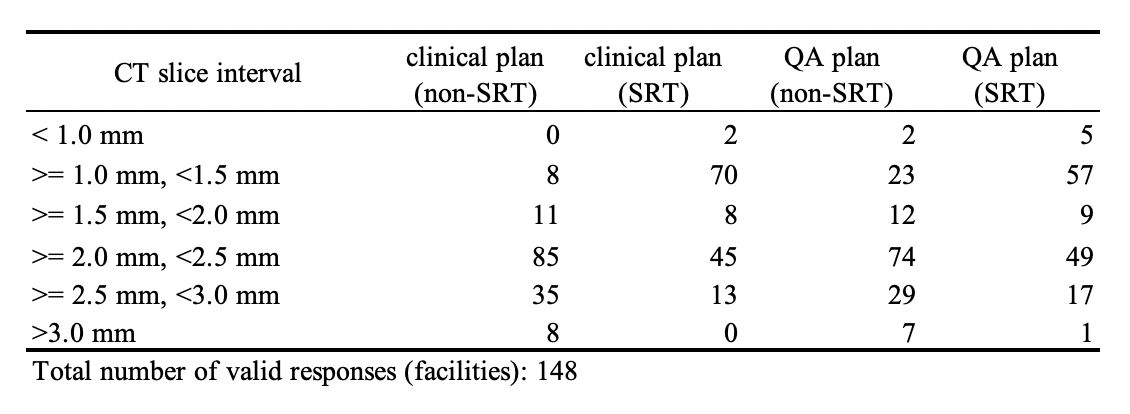
**

**Table S11. Most used dose calculation grids (Q13)**

**
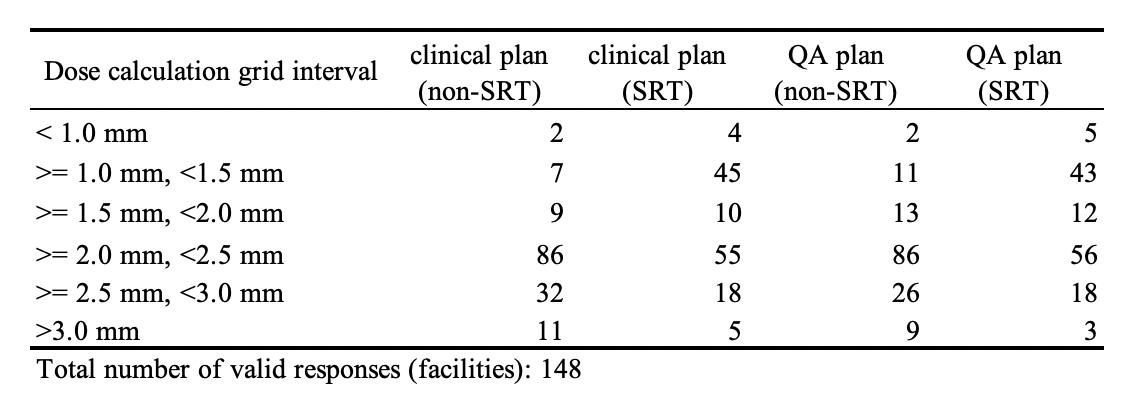
**

**Table S12. Most used gantry spacing resolution for a dose calculation (Q14)**

**
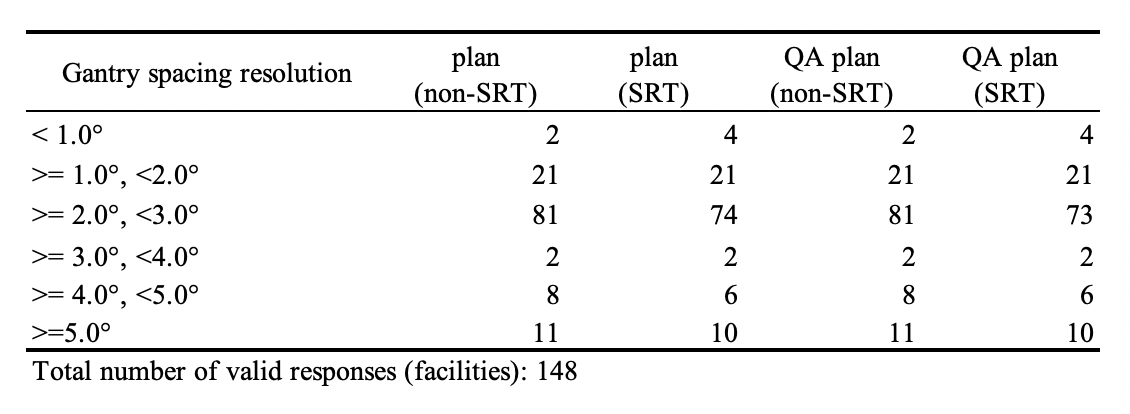
**

**Table S13. Clinically tolerable number of static fields in IMRT planning (Q15)**

**
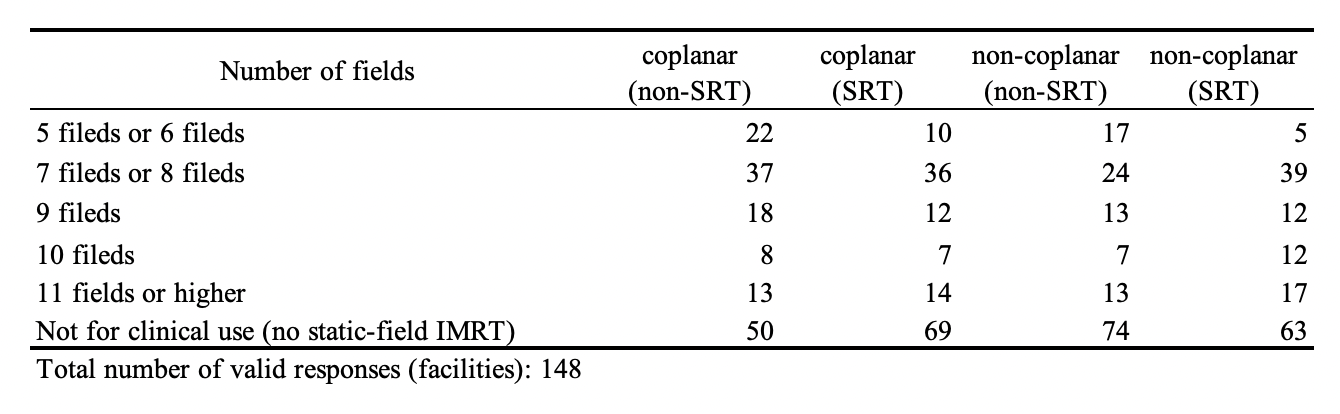
**

**Table S14. Clinically tolerable number of arc fields in VMAT planning (Q16)**

**
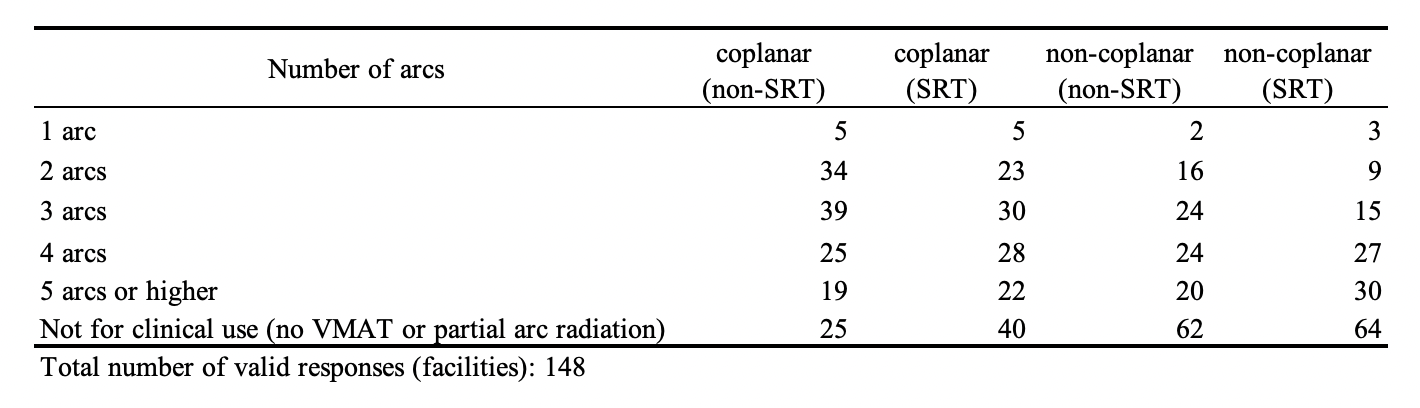
**

**Table S15. Clinically most used beam energy for the tumor targeted region (Q17)**

**
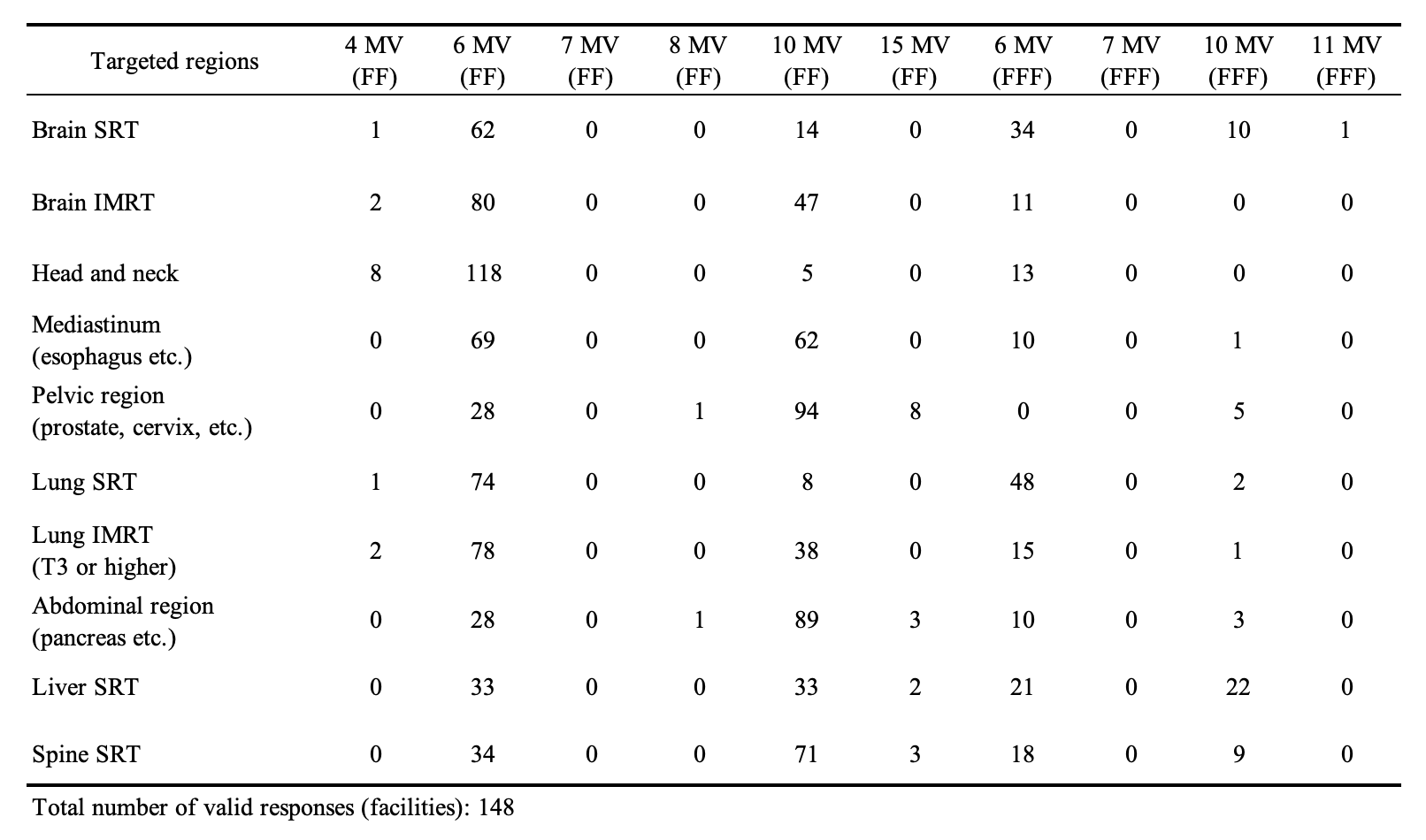
**

**Table S16. Acceptable grounds for treatment approvement (Q18)**

**
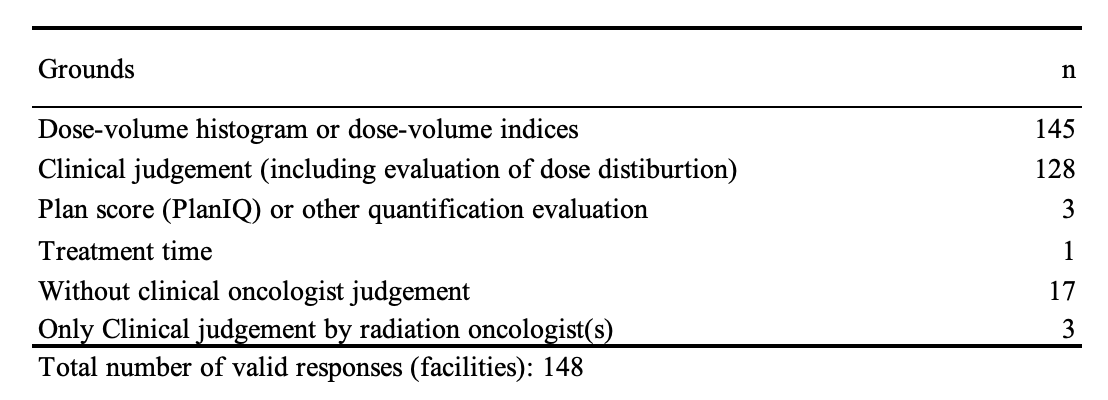
**

**Table S17. Measurement devices for point absorbed dose in the region of interest (Q19)**

**
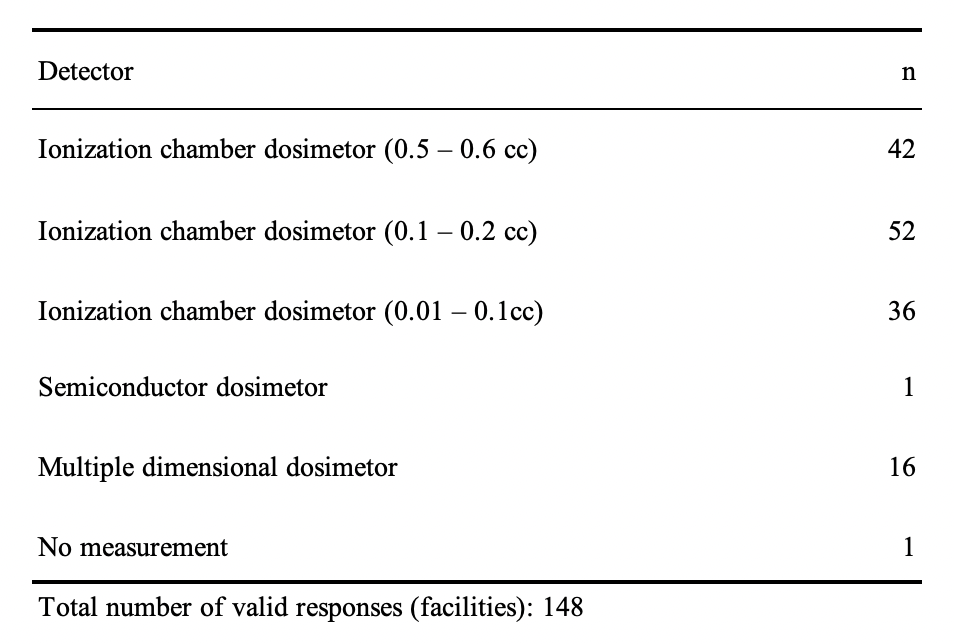
**

**Table S18. Measurement devices for dose distribution (Q20)**

**
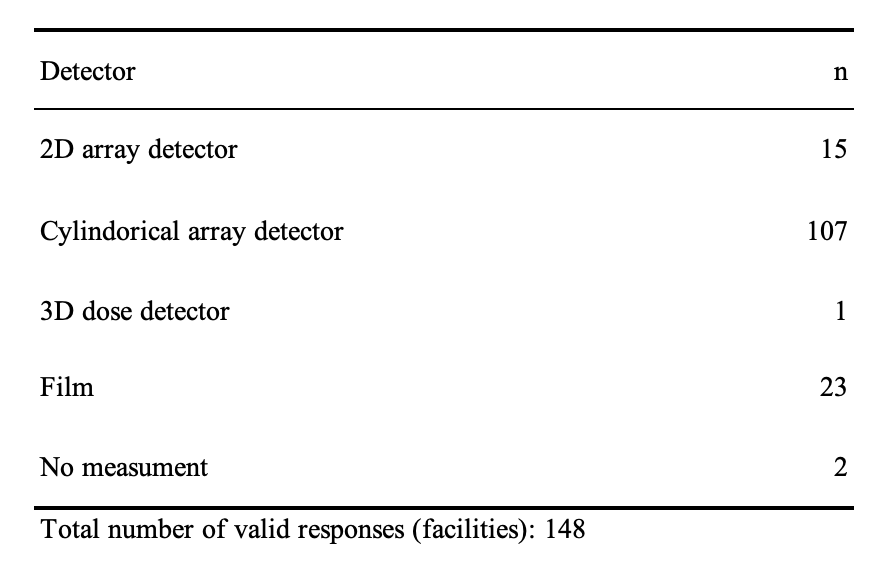
**

**Table S19. Measurement devices for radiation intensity (fluence map) (Q21)**

**
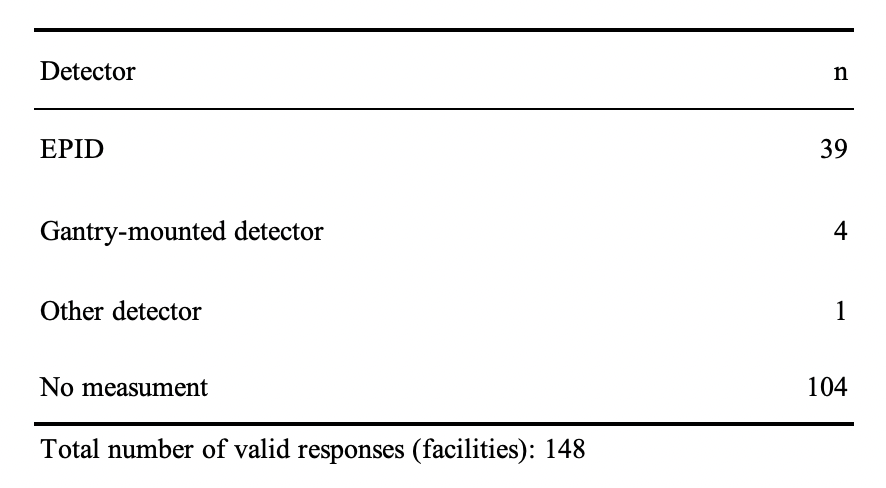
**

**Table S20. Mainly used measurement method for patient-specific QA (Q22)**


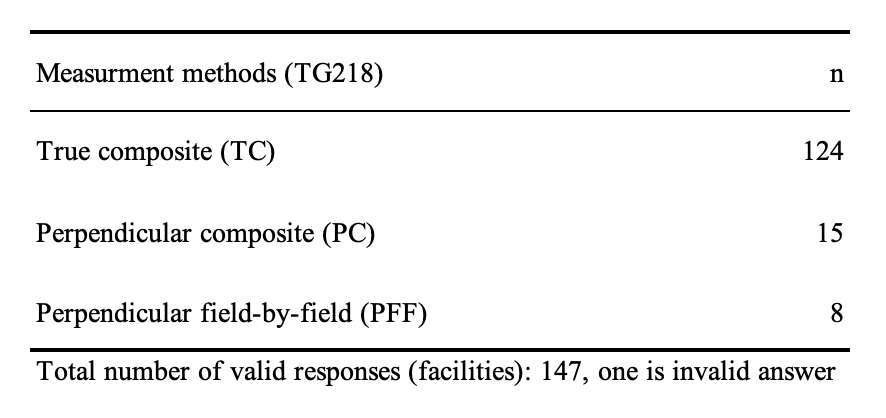


**Table S21. Predicted patient-inner dose distribution consideration (Q23)**

**
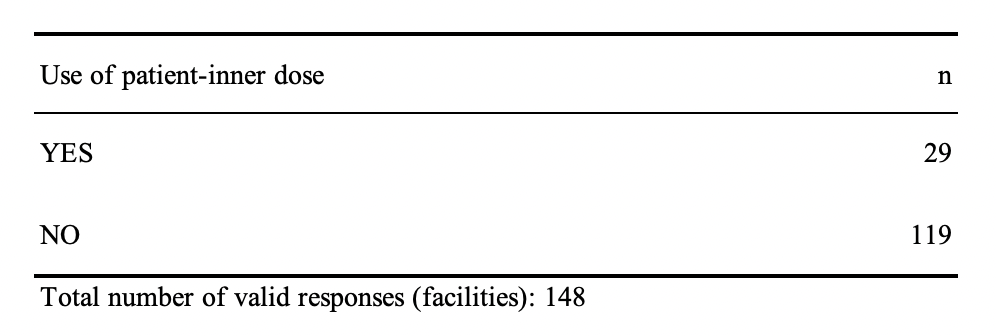
**

**Table S22. Software for predicted patient-inner dose evaluation (Q24)**

**
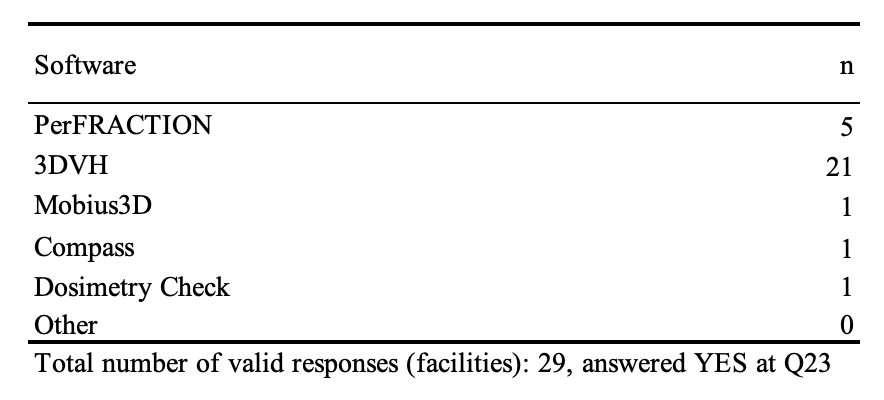
**

**Table S23. Clinical point of interest for the measurement of patient-specific QA(Q25)**

**
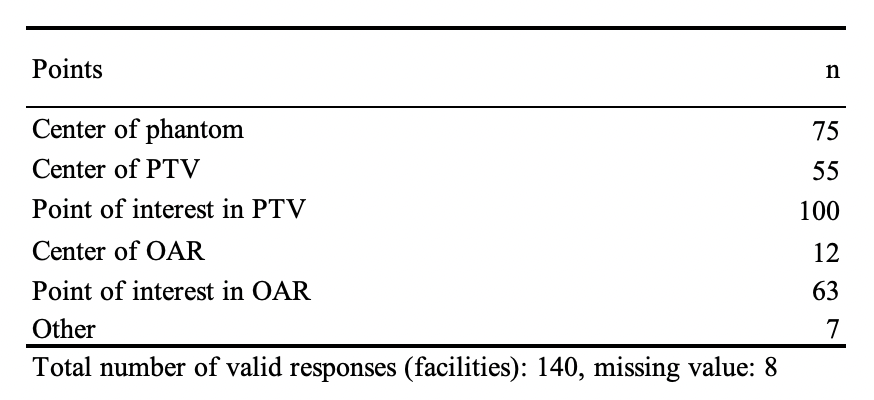
**

**Table S24. Calculation dose for the measurement of patient-specific QA (Q26)**

**
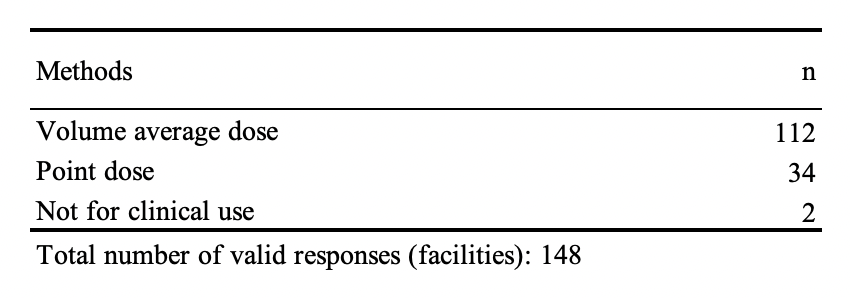
**

**Table S25. Most used phantoms for the measurement of patient-specific QA (Q27)**

**
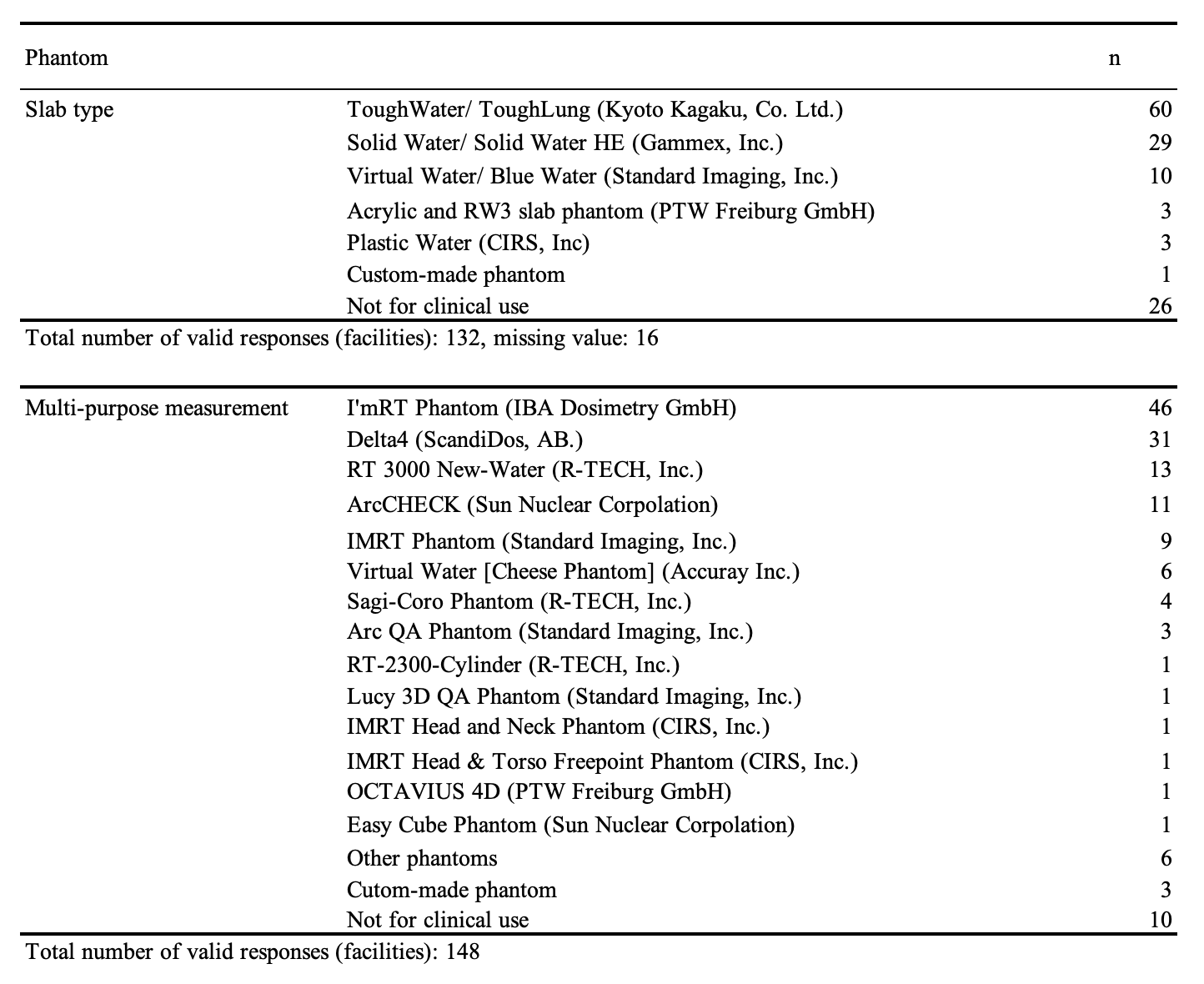
**

**Table S26. Clinically applied method to correct water equivalent material (Q28)**

**
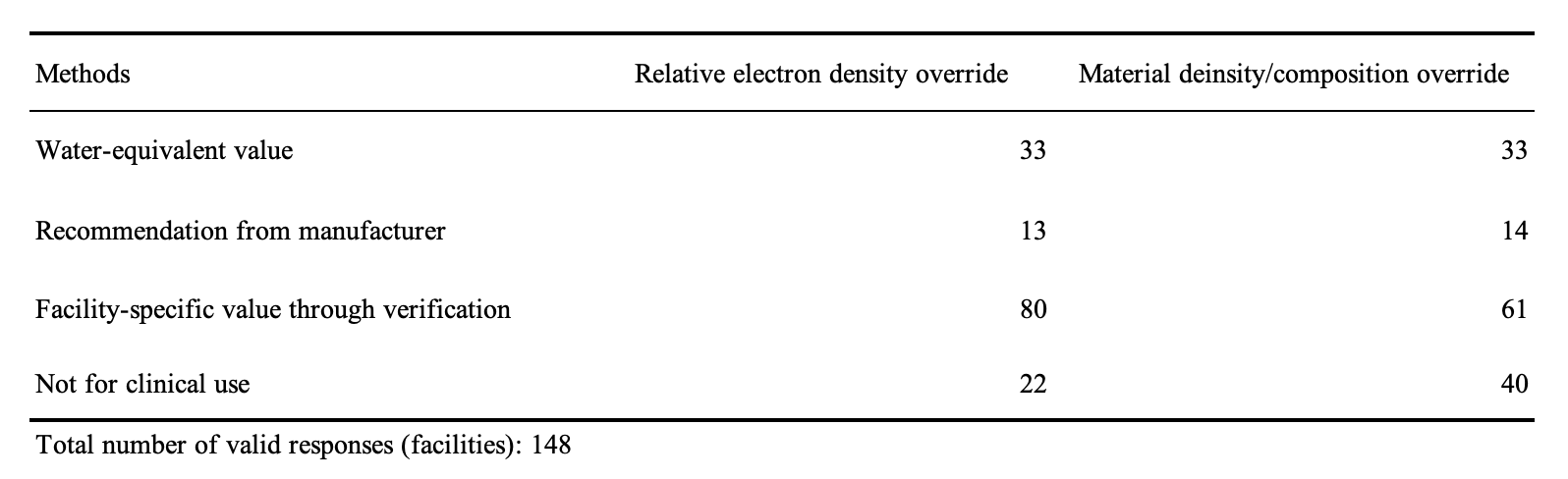
**

**Table S27. Additional auxiliary equipment/device/fixture for dose calculation (Q29)**

**
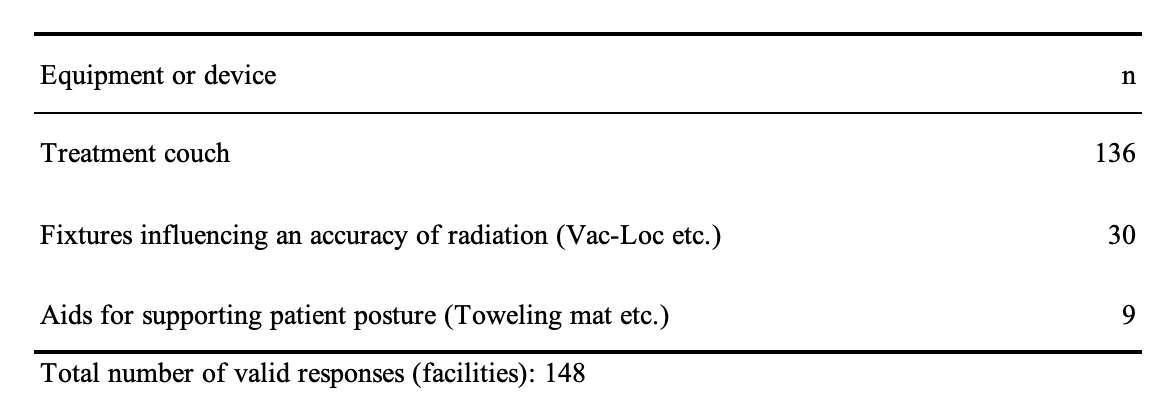
**

**Table S28. Set-up method for the measurement of patient-specific QA (Q30)**

**
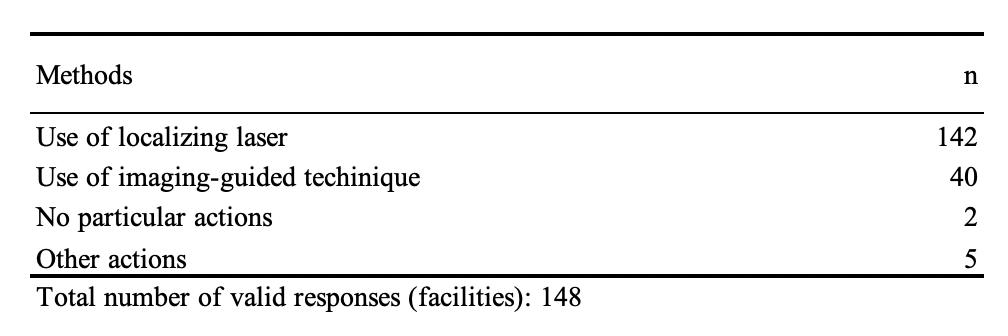
**

**Table S29. Consideration of multi-dimensional detector without normalization (Q31)**

**
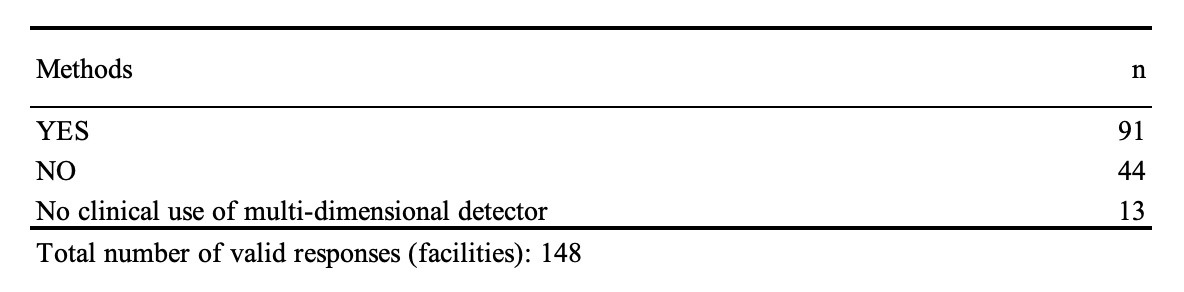
**

**Table S30. Items for mainly evaluating the actual dose distribution (Q32)**

**
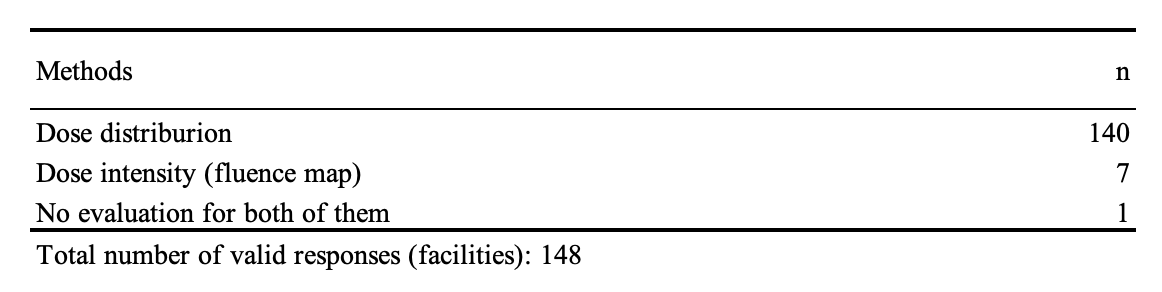
**

**Table S31. Evaluation criteria for dose difference (DD) (Q33)**

**
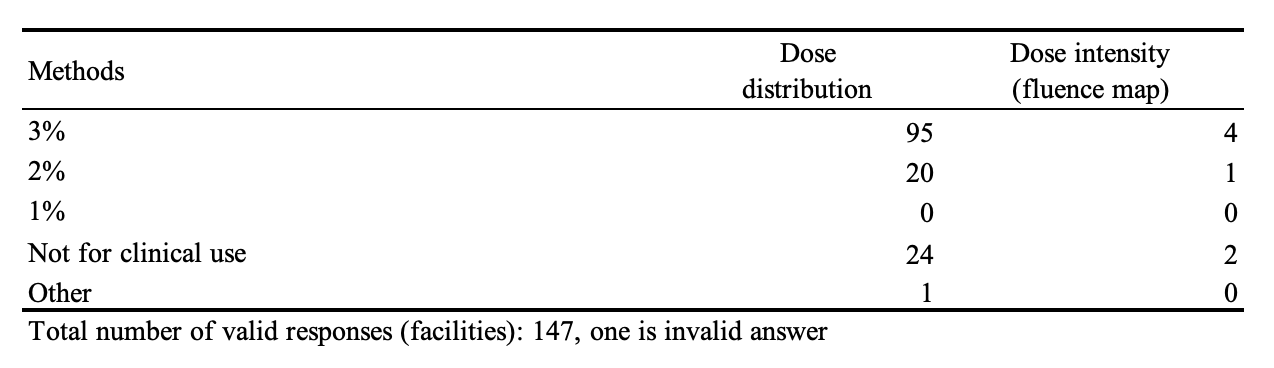
**

**Table S32. Evaluation criteria for dose-to-agreement (DTA) (Q34)**

**
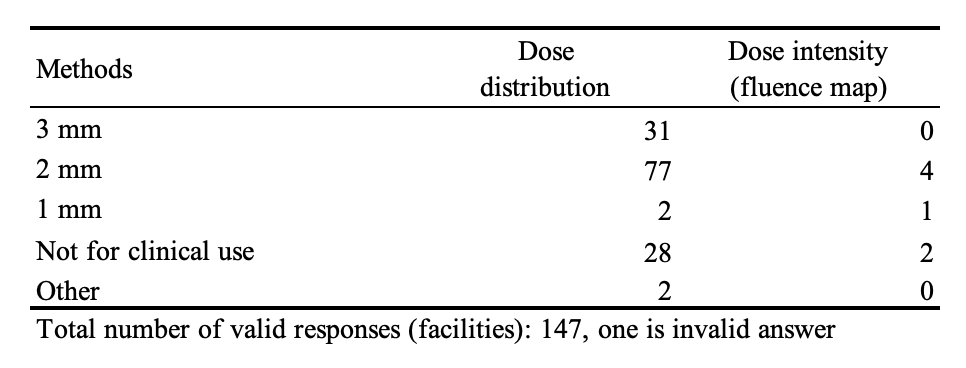
**

**Table S33. Evaluation criteria for gamma analysis (Q35)**

**
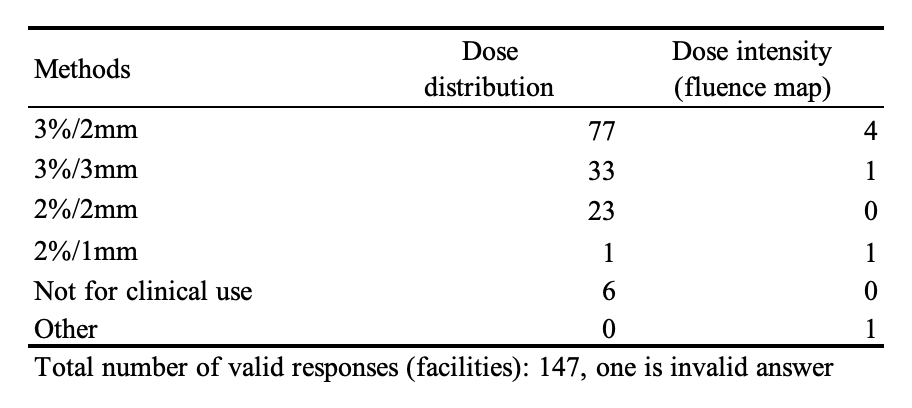
**

**Table S34. Evaluation threshold for gamma analysis (Q36)**

**
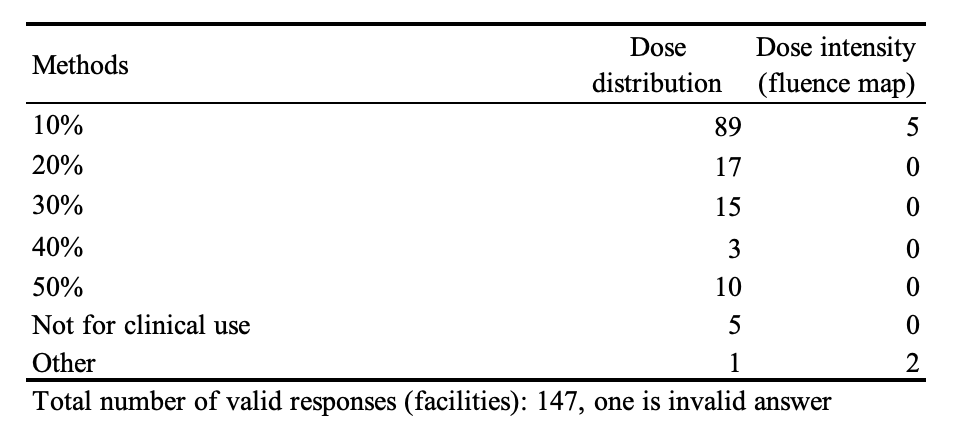
**

**Table S35. Evaluation base dose for gamma analysis (Q37)**

**
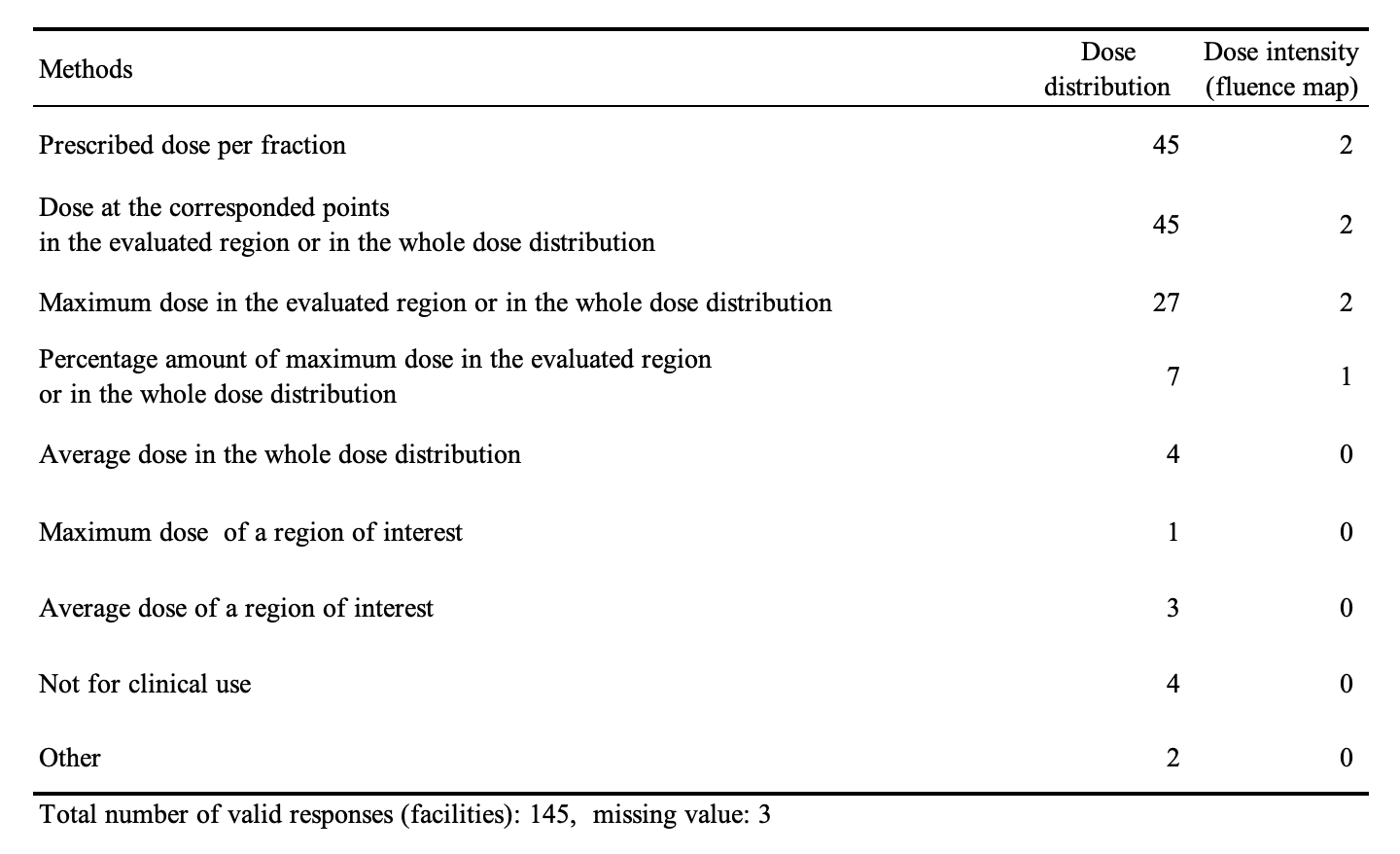
**

**Table S36. Evaluation pass rate for gamma analysis (Q38)**

**
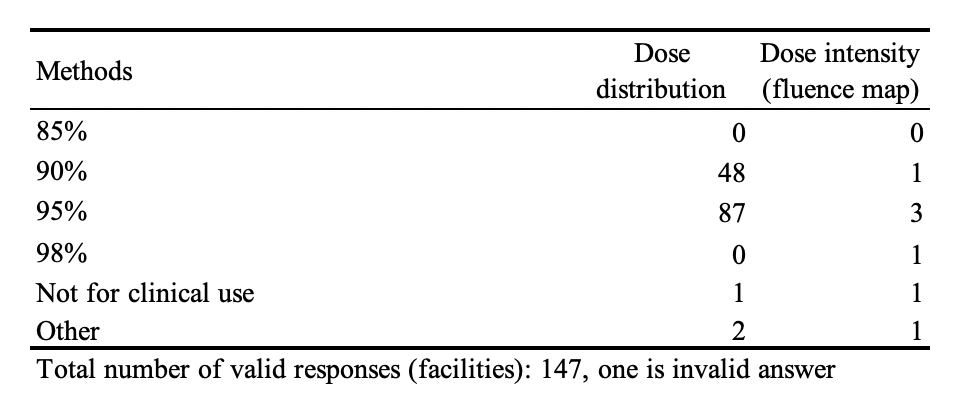
**

**Table S37. Evaluation criteria for predicted patient-inner dose (Q39)**

**
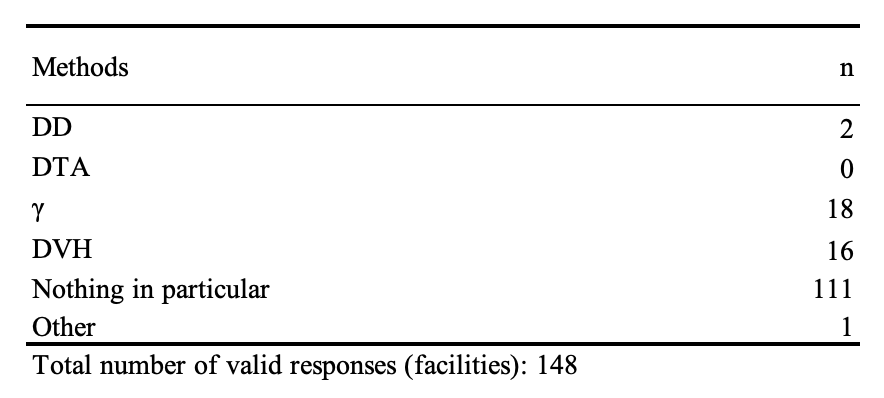
**

**Table S38. References for the measurement of patient-specific QA (Q40)**

**
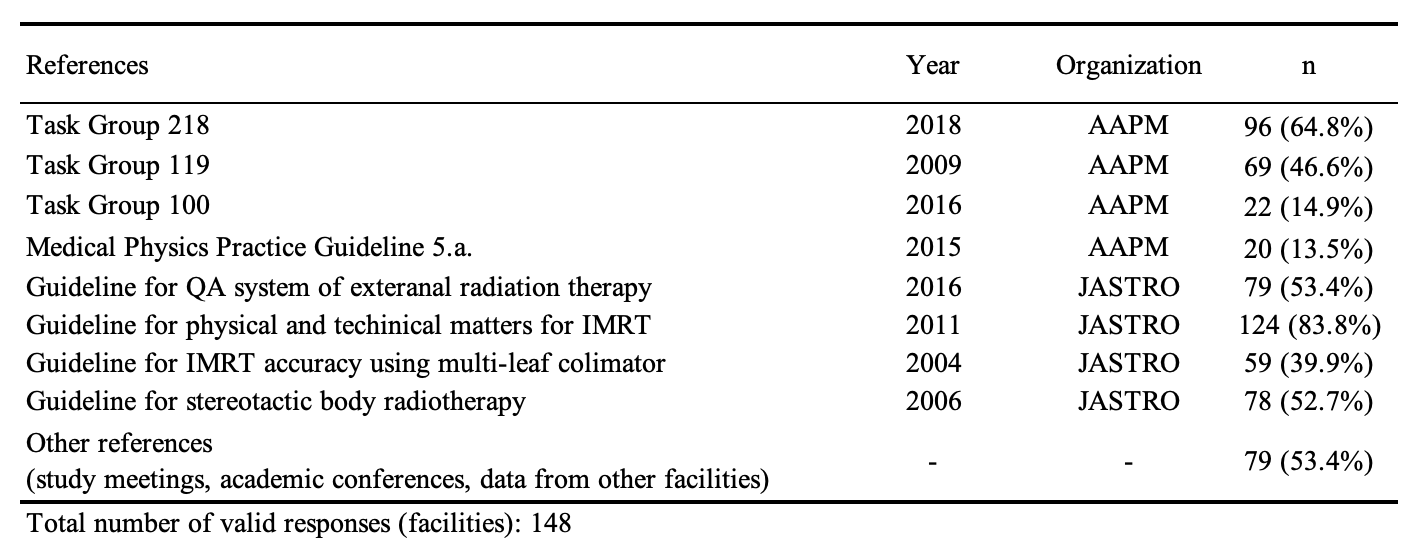
**

Abbreviation: AAPM: The American Association of Physicist in Medicine, JASTRO: Japanese Society for Radiation Oncology.

**Table S39. Tolerance/action level against the patient-specific QA (Q41) considerations**

**
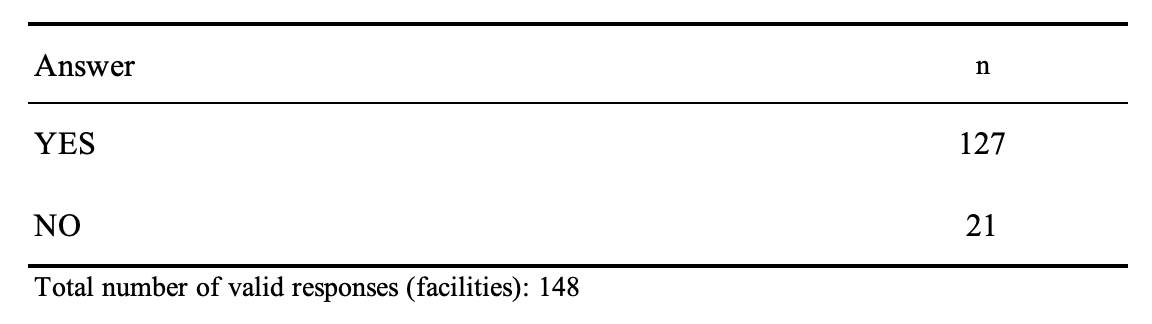
**

**Table S40. Criteria for the tolerance level (Q42)**

**
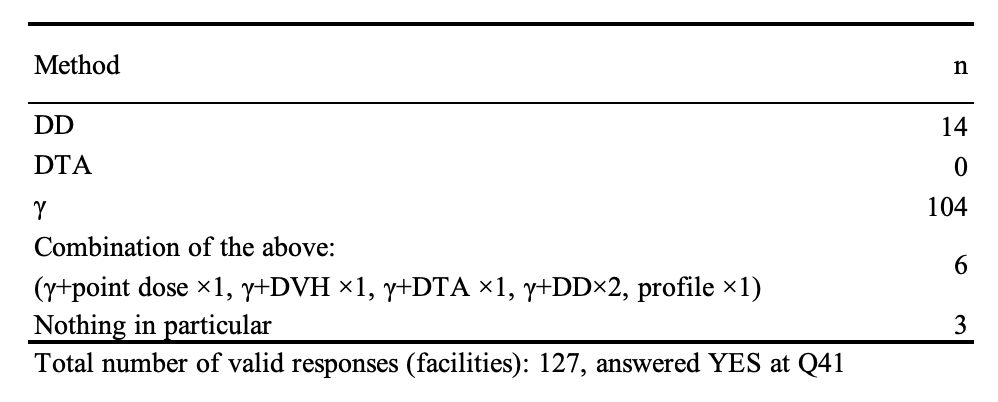
**

**Table S41. Criteria for the action level (Q43)**

**
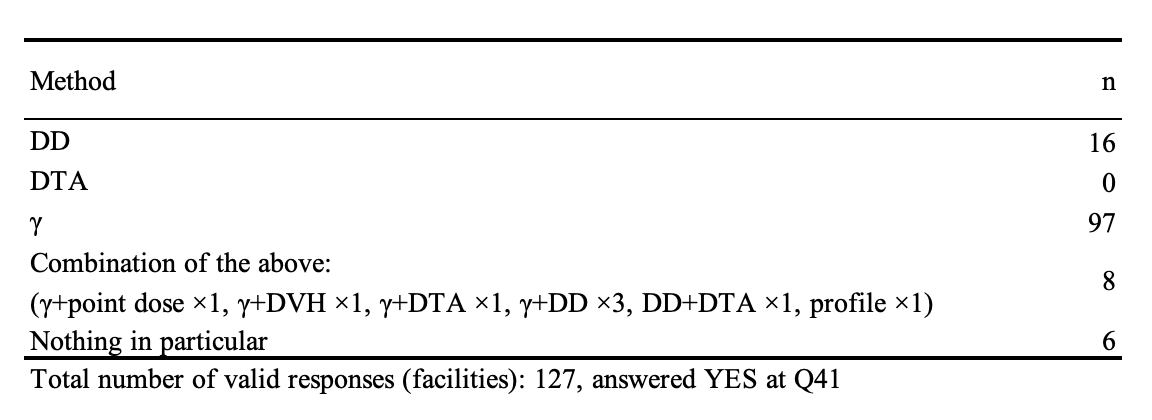
**

**Table S42. Criterion value for the action level in gamma analysis (including multiple criteria) (Q44)**

**
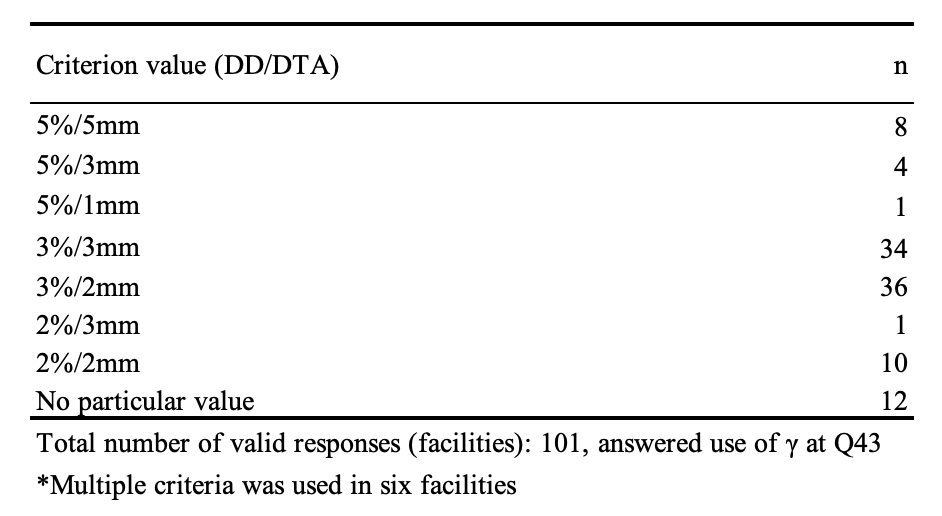
**

**Table S43. Criterion value for the action level in DD analysis (Q44)**

**
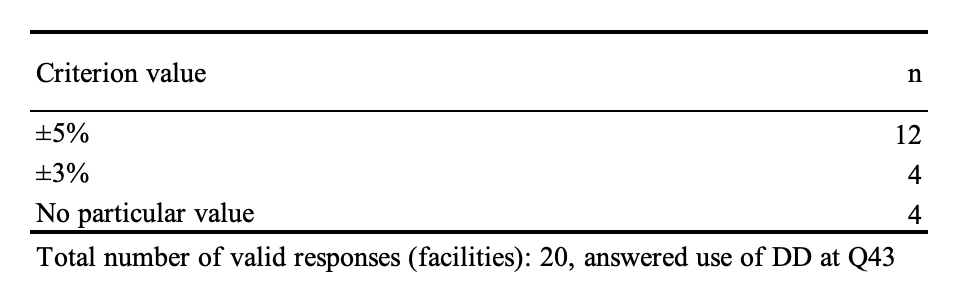
**

**Table S44. Reconsidering the tolerance/action level for PSQA (Q45)**

**
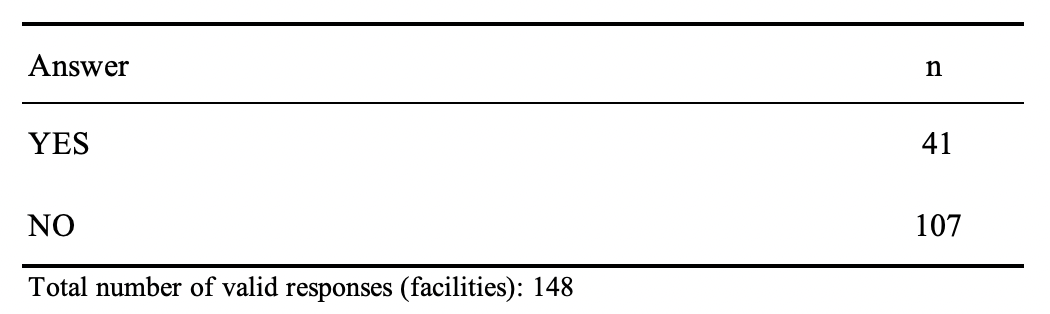
**
